# Supplementary material for: Common attributes in retired professional cricketers that may enhance or hinder quality of life after retirement: a qualitative study
Source: BMJ Open. 2017 Jul 26;7(7):e016541. doi: 10.1136/bmjopen-2017-016541 (PMC5642649; doi:10.1136/bmjopen-2017-016541)
Supplement: Supplementary Appendix 1 [file bmjopen-2017-016541supp001.pdf]

Supplementary Appendix 1 Semi-structured interview guide

1. Can you describe any physical activity, exercise or sport that you currently take part in?
2. Has that remained fairly constant since you retired from cricket or has it changed over the years?
3. Have you played cricket again since retiring? Why/why not?
4. What was your motivation for playing cricket?
5. Are you as physically active as you would like to be? If no, why not? How does this make you feel?
6. What is your motivation for taking part in physical activity/exercise/sport?
7. How important is being physically active to you? (If important, why is it important? / If not important, has it always been this way?)
8. Does the type of physical activity that you do matter to you, or would you be satisfied taking part in any form of physical activity?<br>(ask about specific forms of exercise that they find *dissatisfying* and why)
9. What physical activity goals are you currently trying to achieve, if any?
10. What are the barriers or challenges, if any, that impact on your ability to be physically active?
11. Do you think that retired cricketers face the same challenges with being physically active as the general population, or are they unique or different in some way?
12. Some retired cricketers become physically inactive, what advice would you give to help them maintain a physically active lifestyle after retiring from cricket?
13. If you wanted to increase your physical activity levels, what do you think would help you to do so?
14. Can you describe any positive or negative impacts that your previous participation in cricket has had upon your current physical activity patterns?
15. If you hadn’t played professional cricket, do you think that you would be more or less active, than you currently are?
16. Does your current ability to participate in physical activity impact upon your quality of life? If yes, in what ways? If no, why not?
17. Overall how satisfied are you with your current quality of life?<br>Do you think that this is related to your past career in cricket?
18. Is there anything more you would like to add about your experiences with physical activity after retiring from professional cricket?
