# Supplementary material for: Common attributes in retired professional cricketers that may enhance or hinder quality of life after retirement: a qualitative study
Source: BMJ Open. 2017 Jul 26;7(7):e016541. doi: 10.1136/bmjopen-2017-016541 (PMC5642649; doi:10.1136/bmjopen-2017-016541)
Supplement: Author's manuscript [file bmjopen-2017-016541.draft_revisions.pdf]

BMJ Open

# Quality of life following retirement from professional cricket: retired players' perspectives

| | |
|---|---|
| Journal: | *BMJ Open* |
| Manuscript ID | bmjopen-2017-016541 |
| Article Type: | Research |
| Date Submitted by the Author: | 21-Feb-2017 |
| Complete List of Authors: | Filbay, Stephanie; University of Oxford, Arthritis Research UK Centre for Sport, Exercise & Osteoarthritis, Nuffield Department of Orthopaedics, Rheumatology & Musculoskeletal Sciences<br>Bishop, Felicity; University of Southampton, Psychology<br>Peirce, Nicholas; Loughborough University, National Centre for Sport and Exercise Medicine & National Cricket Performance Centre<br>Jones, Mary; University of Oxford, Arthritis Research UK Centre for Sport, Exercise & Osteoarthritis, Nuffield Department of Orthopaedics, Rheumatology & Musculoskeletal Sciences<br>Arden, Nigel; University of Oxford, Arthritis Research UK Centre for Sport, Exercise & Osteoarthritis, Nuffield Department of Orthopaedics, Rheumatology & Musculoskeletal Sciences |
| <b>Primary Subject Heading</b>: | Sports and exercise medicine |
| Secondary Subject Heading: | Qualitative research |
| Keywords: | SPORTS MEDICINE, Osteoarthritis, PAIN MANAGEMENT, QUALITATIVE RESEARCH, Resilience |
| | |

SCHOLARONE™
Manuscripts

# Quality of life following retirement from professional cricket: retired players' perspectives

**Filbay S.R, Bishop F.L, Peirce N, Jones M.E, Arden N.K.**

Filbay S.R.

Arthritis Research UK Centre for Sport, Exercise & Osteoarthritis, Nuffield Department of Orthopaedics, Rheumatology & Musculoskeletal Sciences, University of Oxford
Botnar Research Centre, Windmill Rd, Oxford UK OX3 7LD
t: 07 414 064 683 | e: stephanie.filbay@uq.net.au | twitter: @stephfilbay

Bishop F.L.

Psychology department, University of Southampton, Southampton, UK. SO17 1BJ
t: +44 (0)23 8059 9020 | e: F.L.Bishop@southampton.ac.uk

Peirce N.

National Centre for Sport and Exercise Medicine & National Cricket Performance Centre, Loughborough University, Leicester, LE11 3TU UK
t: + 44 (0) 1509 228663 | e: nick.peirce@ecb.co.uk

Jones M.E.

Nuffield Department of Orthopaedics, Rheumatology & Musculoskeletal Sciences, University of Oxford
Botnar Research Centre, Windmill Rd, Oxford UK OX3 7LD
t: 01865 223436 | e: mary.jones@oriel.ox.ac.uk

Arden N.K.

Arthritis Research UK Centre for Sport, Exercise & Osteoarthritis, Nuffield Department of Orthopaedics, Rheumatology & Musculoskeletal Sciences, University of Oxford
Botnar Research Centre, Windmill Rd, Oxford UK OX3 7LD
t: +44 (0)1865 737859 | e: nigel.arden@ndorms.ox.ac.uk



For peer review only - http://bmjopen.bmj.com/site/about/guidelines.xhtml

**Word count** 3984 (4000 Word Limit)


# ABSTRACT

## Objectives

Retired professional cricketers shared unique experiences and may possess specific psychological attributes with potential to influence quality of life (QOL). Additionally, pain and osteoarthritis can be common in retired athletes which may negatively impact QOL. However, QOL in retired athletes is poorly understood.

This study explores the following questions from the personal perspective of retired-cricketers: How do retired cricketers perceive and experience musculoskeletal pain and function in daily life? Are there any psychological attributes that might enhance or hinder retired cricketers' QOL?

## Design

A qualitative study using semi-structured interviews, which were subject to inductive, thematic analysis. A data-driven, iterative approach to data coding was employed.

## Setting

All participants had lived and played professional cricket in the United Kingdom and were living in the UK or abroad at the time of interview.

## Participants


For peer review only



For peer review only - http://bmjopen.bmj.com/site/about/guidelines.xhtml

18 male participants, aged a mean 57±11 (range 34 to 77) years had played professional cricket for a mean 12±7 seasons and had been retired from professional cricket on average 23±9 years.

## Results

Fifteen participants reported pain or joint difficulties and all but one was satisfied with their QOL. Most retired cricketers reflected upon experiences during their cricket career that may be associated with the psychological attributes that these individuals shared, including resilience and a positive attitude. Additional attributes included a high sense of body awareness, an ability to self-manage pain and adapt lifestyle choices to accommodate physical limitations. Participants felt fortunate and proud to have played professional cricket, which may have further contributed to the high QOL in this group of retired cricketers.

## Conclusions

Most retired cricketers in this study were living with pain or joint difficulties. Despite this, all but one was satisfied or very satisfied with their QOL. This may be partly explained by the positive psychological attributes that these retired cricketers shared.

## Strengths and limitations of this study

- A strength of this study was the strong themes and consistent patterns that emerged through the interviews and the deep reflection and open, personal experiences that retired-cricketers shared that enriched the findings of this study.
- Since participants had been retired from professional cricket for an average of 23 years, we gained unique insight into longer-term quality of life after professional sport which had not been previously explored.



For peer review only - http://bmjopen.bmj.com/site/about/guidelines.xhtml

- It is not known whether cricket participation contributed to the development of positive psychological attributes or whether these largely pre-existed prior to cricket participation or developed after retirement.
- Limitations include generalisability and the possibility of selection bias, whereby retired cricketers with specific characteristics may be more likely to desire participation in a qualitative interview study.


## Funding

Dr Filbay was awarded a research fellowship from the Arthritis Research UK Centre for Sport, Exercise and Osteoarthritis to support this research. Dr Arden and Mary Jones have received an unrestricted research grant from the England and Wales Cricket Board. The parent cross-sectional study from which participants were recruited received funding from the Arthritis Research UK Centre for Sport, Exercise and Osteoarthritis, as well as the England and Wales Cricket Board.


## Competing interests

Dr Arden and Mary Jones have received an unrestricted research grant from the England and Wales Cricket Board. Dr Peirce is employed as the Chief Medical Officer of the England and Wales Cricket Board. Dr Filbay and Dr Bishop have nothing to disclose.



For peer review only - http://bmjopen.bmj.com/site/about/guidelines.xhtml

## Introduction

Research surrounding the long-term outcomes of a professional sporting career has largely focused on negative impacts rather than investigating the potential for positive psychological impacts that persist beyond retirement.[1] On retirement, athletes commonly experience negative emotions and reduced life satisfaction while transitioning and adapting to an alternative lifestyle,[2] but very little is known about life after this transition. After retirement, athletes are at increased risk of developing osteoarthritis, predominately due to a history of sport-related injury.[3-6] However, retired athletes may possess psychological attributes that could enhance quality of life (QOL) and facilitate effective coping with pain and musculoskeletal impairment. Indeed, while participating in sport, individuals obtain a range of psychological benefits, including greater confidence, life satisfaction and resilience,[7] which may or may not persist following retirement. Additionally, retired athletes may feel proud and accomplished to have played professional sport which could have further positive impacts on QOL. On the other hand, functional limitations in individuals with high athletic identity could negatively impact QOL if their physical activity desires are not satisfied. These complex possible influences on the QOL of retired athletes are poorly understood.

Professional cricket is all-encompassing, the duration of play exceeds most other sports with formats including entire day matches and test matches lasting up to five consecutive days. In cricket, different playing positions have distinct psychological demands. A batsman facing a ball is under great pressure to perform, one slip of concentration or an incorrect decision can see him out of the game with no further opportunity to contribute positively to the team. A fielder in a slip position maintains prolonged intense focus in preparation for a potential catch with an outcome of success or failure determined by a momentary reaction. The demands of



For peer review only - http://bmjopen.bmj.com/site/about/guidelines.xhtml

cricket contrast most other team sports where players have greater opportunity to correct performance and ongoing involvement within a game. Thus, professional cricketers face unique competitive demands that require advanced psychological and behavioural control to manage stress and perform under pressure.[8 9] The ability to deal with such stressors will in part, determine the success of a professional cricketer. Psychological attributes associated with a successful cricket career include resilience, mental toughness, self-belief, optimism, coping with adversity and confidence.[10-12] These attributes enable professional cricketers to overcome performance-inhibiting negative emotions, thrive in high pressure situations and maintain strong self-esteem and confidence irrespective of performance. Professional cricketers may experience a wide variety of injuries during their playing career[13] placing a cricketer at high risk of developing post-traumatic osteoarthritis.[3-6] The psychological attributes that retired cricketers possess have potential to benefit other aspects of life and reduce the impact of osteoarthritis following retirement from cricket.

We undertook a qualitative study to provide an in-depth analysis of life after retirement from professional cricket, from the personal perspective of retired-cricketers. The primary aim was to explore physical activity after retirement from professional cricket. The study also captured participants' broader perspectives regarding QOL and prominent themes linking QOL to professional cricket emerged during the initial inductive analysis. This paper focuses on these emergent themes, which were explored while considering two sensitising questions: How do retired cricketers perceive and experience musculoskeletal pain and function in daily life? Are there any psychological attributes that might enhance or hinder retired cricketers' QOL? A complementary manuscript will address physical activity after retirement from professional cricket.



For peer review only - http://bmjopen.bmj.com/site/about/guidelines.xhtml

## Methods

### *Ethical considerations*

This study has been approved by Medical Sciences Inter-divisional Research Ethics Committee (IDREC), University of Oxford (reference number R45197/RE001).

### *Participants and recruitment*

Participants were purposively sampled from a larger cohort study of 187 retired professional English cricketers who recently completed a cross-sectional questionnaire collecting information on demographics, playing history, current pain, medical history (including doctor diagnosed osteoarthritis, arthroscopic surgery and joint replacement surgery) and injury history (Jones et al. *under review*). In order to facilitate a balanced exploration of physical activity behaviours, barriers, and facilitators, we purposively sampled individuals based on their responses to two questions from the cross-sectional study assessing perceived impact of previous cricket participation upon current physical activity levels: i) Strongly agree or agree that participation in cricket has resulted in an *increase* in current physical activity level (n=46, 42%), or ii) Strongly agree or agree that participation in cricket has resulted in a *decrease* in current physical activity levels (n= 27, 25%).

Only individuals from the larger cross-sectional study who indicated a willingness to participate in future sport-related research were invited (by email) into the current study. Among those expressing an interest in this study, participants were then purposively sampled to ensure men of a range of ages were interviewed. Interviews were conducted until the point



For peer review only - http://bmjopen.bmj.com/site/about/guidelines.xhtml

of data saturation, defined a priori as the point at which no new themes were identified from four consecutive interviews (two from participants with reduced physical activity and two from participants with increased physical activity). After which, additional interviews were performed as deemed appropriate to explore ideas and themes in greater detail after following the semi-structured interview guide. These final interviews were also used to affirm data saturation. If new themes emerged from these final interviews, additional interviews would be conducted until data saturation is satisfied.

A total of 42 email invitations were sent to eligible participants, 19 emails received no response, 2 individuals declined to participate, 2 individuals were unavailable due to overseas travel and 1 individual indicated a willingness to participate but did not respond to further correspondence. Data saturation was achieved by the 14$^{th}$ interview. An additional four interviews were carried out after data saturation and no new themes emerged. Data from all 18 interviews were used for analysis.

***Interviews***

Audio recorded semi-structured telephone interviews were performed by S.R.F. Informed verbal consent was obtained from each participant prior to taking part. Interviews were transcribed verbatim by a research assistant. All transcripts were de-identified during transcription, and an alias was assigned to each participant. The 18 interviews were on average 26 minutes in length (ranging from 18 to 37 minutes).



For peer review only - http://bmjopen.bmj.com/site/about/guidelines.xhtml

A semi-structured interview guide was used to assure that key areas of interest were covered while enabling the researcher to adapt the interview guide to elicit relevant and rich information from respondents by probing and prompting for additional details.[14] The interview guide contained open-ended questions to provide participants with the greatest opportunity to consider personal perspectives and experiences (Appendix 1). The interview guide was iteratively adapted throughout the interviews to incorporate any additional issues of importance to respondents (for example, by adding a question to explore their relationship with cricket post-retirement). Interviewees were given the opportunity to contribute any additional information at the end of the interview.

***Analysis procedure***

An inductive thematic approach was used[15 16] facilitated by NVivo version 11 software.[17] Following each interview, the interviewer summarised and reflected upon initial ideas in a study journal. Transcripts were read and re-read with accompanying audio to identify all information relevant to the research aims.[18] An iterative approach to data coding was employed. All data that might be relevant to the aims of the study were coded into multiple categories to be refined and analysed for themes in subsequent stages of analysis.[15] During the earlier stages of analysis, no attention was given to the prevalence of themes and coding was performed diversely and inclusively, withholding further interpretation. In line with an inductive thematic approach, coding was data-driven, performed without reference to a pre-existing coding structure and with little engagement with literature to avoid sensitization to specific themes.[15 18]



For peer review only - http://bmjopen.bmj.com/site/about/guidelines.xhtml

During the later stages of analysis, the data was further analysed for repeated patterns, codes were sorted into a hierarchical structure representing themes and subthemes, overlapping themes were merged, and those outside the scope of the current study were filed separately. These themes and sub-themes were repeatedly reviewed and refined to confirm external heterogeneity and internal homogeneity within themes and to ensure an accurate representation of the entire dataset.[15 19] During this stage, all transcripts were re-read and the study journal was revisited to check that themes accurately reflected the key issues discussed by participants. A selection of six transcripts representing participants with diverse physical activity patterns were analysed by a second investigator (F.L.B) blinded to the coding structure developed by the first author (S.R.F). A meeting was then held to discuss key themes, and a high level of agreement was achieved between investigators. Key themes and sub-themes will be described with reference to participant quotes.[15 19]

## Results

### *Participant characteristics*

The 18 participants who took part in the interviews were all male, aged a mean 57±11 (range 34 to 77) years, played professional cricket in the United Kingdom for a mean 12±7 (range 2 to 25) seasons and had been retired from professional cricket for an average 23±9 (range 7 to 38) years. The majority were overweight by World Health Organisation (WHO) criteria[20] (n=12, 67%) and three participants (17%) were classified as obese. Current joint pain or difficulties were common (n=15, 83%), 10 people (56%) had been told by a doctor that they had osteoarthritis, 12 participants (67%) had received at least one joint surgery and 5 participants (28%) had received one or more total joint replacement. More than two thirds



For peer review only - http://bmjopen.bmj.com/site/about/guidelines.xhtml

(n=14, 78%) reported a past history of injury leading to at least one month of reduced participation in exercise. Full participant characteristics are reported in Table 1.

[Insert Table 1]

***Pain and musculoskeletal function***

Fifteen retired cricketers described experiencing current pain and limitations with musculoskeletal function. For most individuals, this prevented them for taking part in specific activities, like running and recreational sports. Although this was described as frustrating by some, participants had made lifestyle modifications to accommodate these impairments and very few described a negative impact upon QOL.

> *Jim: 'I've had 13 operations over the years because of cricket injuries and resulting stuff from that and I'm just restricted in what I can do really. ..it doesn't make me feel, nothing really, as long as I can do the little bits I can I'm quite happy.'*

> *Sam: 'So there are little bits that frustrate me but I mean, I'm nearly 60 so you know, your body does get worn out if you do something as extreme as play a professional sport for 17 years, so yeah they're just things that exist, but no, I'm not that bothered.'*

***The ups and downs of professional cricket***

Retired-cricketers described experiencing mental hardship and psychological challenges during their cricket career. These included coming to terms with experiencing more failure than success and difficulty coping with pressure to perform. Almost all participants believed these experiences benefited other areas of life after retirement. Specifically, they described how attributes required to succeed on the cricket field, including mental toughness, confidence, and resilience, equipped them with tools to overcome post-retirement life



For peer review only - http://bmjopen.bmj.com/site/about/guidelines.xhtml

challenges, including managing chronic pain and establishing a successful career outside of cricket.

*Lee 'It was just a constant battle, it was soul destroying at times but out of all that you know; hard work, disappointment, you know, being beaten down by people at times… I think it's given me the mental capacity just to, just get your head down and battle on. I think that's the main thing, you can have bad times in whatever you're doing, and you're going to feel really low, but I think just that mental capacity that you've learnt from cricket, from being a professional sportsmen, to just get back up on your feet and fight another day sort of thing is being part of where I've got to now, you know thanks to that basically.'*

Only one individual felt that the mental aspects of professional cricket had a negative impact upon life after retirement. Dan attributed difficulty coping with adversity and reduced confidence to having played professional cricket.

*Dan: 'I wouldn't say I carry mental scars, but I think it's affected my personality.. ..probably I am less confident having been a professional sportsman than I would have been otherwise, because I think I have been beaten up quite a few times over the years, you know mentally, and it's taken its toll a little bit.'*

***Psychological attributes that may enhance quality of life***

*Resilience and a positive attitude*

Attributes that appeared to enhance QOL in this sample of retired cricketers, included resilience and a positive attitude. Language used throughout the interviews highlighted that almost all retired-cricketers in this study were resilient and had a positive attitude regarding pain and musculoskeletal impairment.

*Dom: 'I would classify myself to be very, very lucky, probably very, very lucky to come out the other side, with just, at the moment, hip injuries that have been cured by hip operations… it's all been extremely positive, from a life style I've reached, yeah.'*

*Ron: 'Keep a strong mind and you will have aliments depending on how much sport you have played and you just have to get on with it.''*



For peer review only - http://bmjopen.bmj.com/site/about/guidelines.xhtml

*Body awareness, acknowledging limitations and self-management*

There were other common attributes amongst the retired cricketers that appeared to have a positive impact upon QOL, these included a heightened sense of body awareness and confidence in self-managing physical ailments. Most participants had made positive adaptations and were mindful of their body's capabilities. Participants adhered to self-imposed activity limits and modified their activity choices on a day to day basis to avoid exacerbation of pain or an acute deterioration in physical function.

> *Fin: '.. you also have a greater and wiser knowledge of what you should, could, and can and can't do. So from a positive point of view you know what you can and can't do and you know what's good for you and your body.'*

> *Gus: 'I like to think that I know what my, what my boundaries are and I can just push them a little bit or to the limit that I think that, that my age will allow me to do... I definitely think that playing cricket made me certainly more aware and being involved in cricket for the amount of time that I had, I certainly have, have more knowledge of what I can and can't do.'*

*Fortunate, proud and grateful to have played professional cricket*

Reflecting upon their past career in cricket, all participants felt fortunate, proud or grateful to have had the opportunity to play professional cricket. All but one individual believed that overall, the positive effects of cricket outweighed the negative, and expressed no regret over playing professional cricket.

> *Fin: 'No regrets what so ever. It was a great life. I had a great time. There were things that, when you come out of that sporting environment into the real world, if you like, and you realise that then its only things that other people dream of doing, is what you've done. So absolutely no regrets what so ever.'*

> *Sam: 'I'd rather have 21 test matches and then that be the end of it, than not have them. You just play the hand your dealt and I never think there's any point having any regrets or bearing any grudges, it is what it is and you know I've had a great life out of it. ...I've made plenty of rubbish decisions, but I wouldn't change that fact, because all that failure and all that success has made me who I am and I'm quite happy being me now.'*



For peer review only - http://bmjopen.bmj.com/site/about/guidelines.xhtml

***Quality of life***

Irrespective of pain and joint difficulties, most retired-cricketers were very satisfied (n=11, 61%) or satisfied (n=6, 33%) with their QOL. No participants expressed that their QOL was impaired due to pain or physical issues. The only individual dissatisfied with his QOL attributed this entirely to current family difficulties.

> Interviewer: Overall how satisfied are you with your current quality of life?
>
> *Wes: '110%, cannot believe my luck. ..I am spoilt rotten, but I mean there's no point in being in a brilliant position, which I am, if you don't appreciate it, I mean I can see lots of people who don't know how lucky they are, well, I am glad to say I do.*
>
> *Sam: 'I am absolutely delighted with it, I love it. I'm in a really good place mentally… things couldn't be better.'*
>
> *Leo: 'Oh brilliant, I'm just very, very lucky, absolutely brilliant.'*
>
> *Ken: 'I am very happy with it actually, and have been for a number of years.'*
>
> *Joe: 'Good, yeah I'm very happy.'*
>
> *Guy: 'Oh on a scale, I would say 8/9 out of 10; I think I am very satisfied.'*
>
> *Gus: 'Well I am very happy, very happy and pleased with my quality of life.'*
>
> *Dom: 'Very happy. ..very happy with where I am physically and mentally in what I do and what I want to do, yes.'*
>
> *Ned: 'Oh generally, you know, very happy really, I think you know keeping things in perspective of, you know of how I am, my age, you know, what I've got, you know I can't complain really. I am really pretty pleased with the way things are.'*

***Summary of key themes***

Figure 1 provides a conceptual flow diagram summarising the main findings and providing an overview of how the key themes may interact to make a positive contribution to QOL after retirement.



For peer review only - http://bmjopen.bmj.com/site/about/guidelines.xhtml

[Insert Figure 1 here]

## Discussion

Current pain and physical limitations were common in this group of retired professional cricketers. Despite this, almost all retired cricketers were satisfied or very satisfied with their QOL. One potential explanation for this counterintuitive finding is the common psychological attributes that we found these individuals to possess, including resilience, a positive attitude, heightened body awareness, acknowledgement of physical limitations, and an ability to modify activity choices to effectively cope with fluctuations in pain and musculoskeletal function. Reflecting upon their past cricket career, participants felt very grateful and fortunate to have played professional cricket, and all but one felt the positive impacts of a career in cricket outweighed any negatives. This feeling of pride and accomplishment has further potential to positively influence QOL in this group of retired cricketers, irrespective of pain and physical impairment.

### *Quality of life and flourishing*

We found that all but one retired-cricketer was satisfied with his QOL. Flourishing is a concept that represents an optimal QOL where an individual excels emotionally, psychologically and socially. In 2013, 20% of a representative sample of the UK general population were found to be ‘flourishing’ (possessing 9 of 10 signs of positive wellbeing including resilience, optimism, competence, self-esteem and meaning).[21] Retired cricketers who took part in the interviews demonstrated many attributes consistent with the definition of



For peer review only - http://bmjopen.bmj.com/site/about/guidelines.xhtml

flourishing. It is possible that a greater proportion of retired-cricketers 'flourish' in later life compared with the general population despite a higher prevalence of pain and osteoarthritis, although further research is necessary to explore this possibility. Notably, most conventional QOL outcome measures are driven by pain and physical impairment, where the presence of pain and physical disability will result in a reduced QOL score, irrespective of the impact of pain or disability upon the individual.[22] Evaluation of the psychometric properties of QOL measures in retired sporting populations is warranted. The Flourishing Scale[23] or a QOL measure not driven by pain and function (such as the WHO-QOL[24]) may be preferable for use in retired sporting populations.

***Resilience and associated positive psychological attributes***

Resilience refers to responding to adversity with positive adaptation, and in doing so, restoring an internal sense of emotional and psychological balance.[25,26] Retired-cricketers in the present study shared resilient attributes, this was highlighted by their ability to maintain a positive attitude despite pain and physical limitations and positively adapt their activity choices to effectively cope with fluctuations in pain and physical function. Prior research has found that athletes possess greater resilience and optimism than non-athletes[27] and successful cricketers are likely to be more resilient than less successful cricketers.[10,12] A high level of resilience is associated with increased QOL in a wide variety of disease-specific and chronic pain populations.[28-35] Resilient individuals also demonstrate more adaptive coping styles and better musculoskeletal pain adjustment.[36,37] Considering sporting injury is a risk factor for osteoarthritis, interventions to build resilience within a sporting setting[38] may have benefits that extend beyond success on the sporting field, with potential to enhance QOL in later life.



For peer review only - http://bmjopen.bmj.com/site/about/guidelines.xhtml

Despite these benefits, who is likely to become resilient and why is poorly understood.[39] The relationship between resilience and professional cricket is likely multifaceted. Exposure to the demands of cricket from a young age, including repetitive failure, disappointment, stress and adversity may facilitate the development of resilience. On the other hand, resilience may be influenced by a range of non-cricket related factors, and resilient individuals may be most likely to reach professional status resulting in a high level of resilience in professional cricketers. It is also possible that adversities faced during transition from sport and after retirement contributed to the high level of resilience in this group of retired cricketers.

***Cricket reflection***

Accomplishment refers to success, achievement or mastery at the highest possible level[40] and has been recognised as an important component of wellbeing.[41] Considering all retired-cricketers in this study were fortunate, proud or grateful to have played professional cricket it is possible that this accomplishment further contributed to the high QOL described by these individuals. A retired athlete's sense of accomplishment and fulfilment through past participation in sport is too often overlooked in research, despite potential to positively influence QOL.

***Study strengths and potential limitations***

It is not known whether cricket participation contributed to the development of positive psychological attributes or whether these largely pre-existed prior to cricket participation or developed after retirement. Prospective longitudinal studies would be required to answer this question. Although participants were asked about their degree of satisfaction with their



For peer review only - http://bmjopen.bmj.com/site/about/guidelines.xhtml

overall QOL, we did not explore personal beliefs about the definition of QOL. Consequently the question pertaining to QOL could have meant different things to different participants. Due to purposive recruitment, individuals reporting uncertainty regarding the impact of cricket upon their physical activity level were not invited into the study, reducing the generalisability of results. It is also possible that the study was subjected to selection bias, whereby retired cricketers with specific characteristics were more likely to desire participation in a qualitative interview study. A strength of this study was the strong themes and consistent patterns that emerged through the interviews resulting in saturation of data at 14 interviews, confirmed by the final four interviews that enabled further complimentary exploration into ideas and important themes. The interviewer was a physiotherapist with experience in building rapport with individuals and capturing issues of importance to an individual. Rapport was further strengthened by the interviewer's familiarity with cricket as a physiotherapist and cricket participant. These factors facilitated deep reflection and open, personal insights from retired-cricketers that enriched the findings of this study.

## Conclusion

Irrespective of chronic joint pain and physical limitations, all but one retired-cricketer in this study was satisfied or very satisfied with their QOL. Retired-cricketers shared common psychological attributes including resilience and a positive attitude that may partly explain the small impact pain and physical limitations appeared to have upon the QOL of these retired cricketers. Additionally, most retired cricketers had a high sense of body awareness and executed self-control to adapt activity choices and accept physical limitations. Participants felt fortunate and grateful to have played professional cricket and expressed that the benefits of cricket participation outweigh any negative impacts. This study highlights



For peer review only - http://bmjopen.bmj.com/site/about/guidelines.xhtml

fruitful areas for future research including i) flourishing in retired athletes despite pain and functional limitations, ii) the potential for positive psychological attributes to develop through sport participation and impact QOL after retirement, and iii) the effect of past accomplishment in sport on QOL across the lifespan. A better understanding of the relationship between sport participation and QOL could assist in promoting physical activity and facilitate strategies to enhance the positive impact of sport upon QOL.

## Acknowledgments

We would like to thank Angus Porter and the Professional Cricketers' Association (PCA) for assisting with recruitment and questionnaire development for the larger cross-sectional study from which study participants were purposively recruited.

## Author contributions

SRF, FLB, NP, NKA conceived and designed this qualitative study. SRF, MEJ recruited participants and extracted data form the cross-sectional cohort. SRF performed all interviews. SRF, FLB participated in the analysis. SRF drafted the first version of the manuscript. All authors contributed in revising the manuscript and gave their final approval of the submitted version.

## Data sharing

To view interview transcripts or additional participant quotes, please contact the corresponding author.

For peer review only



For peer review only - http://bmjopen.bmj.com/site/about/guidelines.xhtml

# References

1. Knights S, Sherry E, Ruddock-Hudson M. Investigating Elite End-of-Athletic-Career Transition: A Systematic Review. Journal of Applied Sport Psychology 2016:1-18.
2. Park S, Lavallee D, Tod D. Athletes' career transition out of sport: a systematic review. International Review of Sport and Exercise Psychology 2013;6(1):22-53.
3. Spector T, Harris P, Hart DJ, et al. Risk of osteoarthritis associated with long-term weight-bearing sports. Arthritis and rheumatism 1996;39(6):988-95.
4. Thelin N, Holmberg S, Thelin A. Knee injuries account for the sports-related increased risk of knee osteoarthritis. Scandinavian journal of medicine & science in sports 2006;16(5):329-33.
5. Gelber AC, Hochberg MC, Mead LA, et al. Joint injury in young adults and risk for subsequent knee and hip osteoarthritis. Annals of internal medicine 2000;133(5):321-28.
6. Gouttebarge V, Inklaar H, Backx F, et al. Prevalence of osteoarthritis in former elite athletes: a systematic overview of the recent literature. Rheumatology international 2015;35(3):405-18.
7. Eime RM, Young JA, Harvey JT, et al. A systematic review of the psychological and social benefits of participation in sport for adults: informing development of a conceptual model of health through sport. International Journal of Behavioral Nutrition and Physical Activity 2013;10.
8. Neil R, Bowles HCR, Fleming S, et al. The experience of competition stress and emotions in cricket. Sport Psychologist 2016;30(1):76-88.
9. Thelwell RC, Weston NJ, Greenlees IA. Batting on a sticky wicket: Identifying sources of stress and associated coping strategies for professional cricket batsmen. Psychology of Sport and Exercise 2007;8(2):219-32.
10. Bull SJ, Shambrook CJ, James W, et al. Towards an understanding of mental toughness in elite English cricketers. Journal of Applied Sport Psychology 2005;17(3):209-27.
11. Weissensteiner J, Abernethy B, Farrow D. Towards the development of a conceptual model of expertise in cricket batting: A grounded theory approach. Journal of Applied Sport Psychology 2009;21(3):276-92.
12. Weissensteiner JR, Abernethy B, Farrow D, et al. Distinguishing psychological characteristics of expert cricket batsmen. Journal of Science and Medicine in Sport 2012;15(1):74-79.
13. Stretch RA. Cricket injuries: a longitudinal study of the nature of injuries to South African cricketers. British Journal of Sports Medicine 2003;37(3):250-53.
14. Jones I, Brown L, Holloway I. *Qualitative Research in Sport and Physical Activity*: SAGE Publications, 2012.
15. Braun V, Clarke V. Using thematic analysis in psychology. Qualitative Research in Psychology 2006;3(2):77-101.
16. Thomas DR. A General Inductive Approach for Analyzing Qualitative Evaluation Data. American Journal of Evaluation 2006;27(2):237-46.
17. Bazeley P, Jackson K. *Qualitative data analysis with NVivo*. Los Angeles: SAGE, 2013.
18. Tuckett AG. Applying thematic analysis theory to practice: A researcher's experience. Contemporary Nurse 2005;19(1-2):75-87.
19. Patton MQ. *Qualitative evaluation and research methods*, 1990.
20. Organisation WH. Obesity: Preventing and managing the global epidemic. Report of a WHO Consultation on Obesity, Geneva,. WHO/NUT /NCD/98 1, Geneva, 1998 3-5 June 1997.
21. Huppert FA, So TTC. Flourishing Across Europe: Application of a New Conceptual Framework for Defining Well-Being. Social Indicators Research 2013;110(3):837-61.
22. Filbay SR. Longer-term quality of life following anterior cruciate ligament injury and reconstruction. PhD Thesis, School of Health and Rehabilitation Sciences, The University of Queensland 2016;1st July 2016.
23. Diener E, Wirtz D, Tov W, et al. New Well-being Measures: Short Scales to Assess Flourishing and Positive and Negative Feelings. Social Indicators Research 2010;97(2):143-56.



For peer review only - http://bmjopen.bmj.com/site/about/guidelines.xhtml

24. Group TW. Development of the World Health Organization WHOQOL-BREF Quality of Life Assessment. Psychological medicine 1998;28(3):551-58.
25. Galli N, Gonzalez SP. Psychological resilience in sport: A review of the literature and implications for research and practice. International Journal of Sport and Exercise Psychology 2015;13(3):243-57.
26. Schinke RJ, Battochio RC, Dube TV, et al. Adaptation Processes Affecting Performance in Elite Sport. Journal of Clinical Sport Psychology 2012;6(2):180-95.
27. Guillén F, Laborde S. Higher-order structure of mental toughness and the analysis of latent mean differences between athletes from 34 disciplines and non-athletes. Personality and Individual Differences 2014;60:30-35.
28. Li MY, Yang YL, Liu L, et al. Effects of social support, hope and resilience on quality of life among Chinese bladder cancer patients: A cross-sectional study. Health and Quality of Life Outcomes 2016;14(1).
29. Ring A, Jacoby A, Baker GA, et al. Does the concept of resilience contribute to understanding good quality of life in the context of epilepsy? Epilepsy and Behavior 2016;56:153-64.
30. Wu WW, Tsai SY, Liang SY, et al. The Mediating Role of Resilience on Quality of Life and Cancer Symptom Distress in Adolescent Patients With Cancer. Journal of Pediatric Oncology Nursing 2015;32(5):304-13.
31. Vitali SA. Finding quality of life despite MS: Harnessing resilience. International MS Journal 2011;17(3):94-99.
32. Frigiola A, Bull C, Wray J. Exercise capacity, quality of life, and resilience after repair of tetralogy of Fallot: A cross-sectional study of patients operated between 1964 and 2009. Cardiology in the Young 2014;24(1):79-86.
33. Manne SL, Myers-Virtue S, Kashy D, et al. Resilience, Positive Coping, and Quality of Life among Women Newly Diagnosed with Gynecological Cancers. Cancer Nursing 2015;38(5):375-82.
34. Yazdi-Ravandi S, Taslimi Z, Saberi H, et al. The role of resilience and age on quality of life in patients with pain disorders. Basic and Clinical Neuroscience 2013;4(1):24-30.
35. Bergman S, Jacobsson LT, Herrstrom P, et al. Health status as measured by SF-36 reflects changes and predicts outcome in chronic musculoskeletal pain: a 3-year follow up study in the general population. Pain 2004;108(1-2):115-23.
36. Hildon Z, Montgomery SM, Blane D, et al. Examining Resilience of Quality of Life in the Face of Health-Related and Psychosocial Adversity at Older Ages: What is “Right” About the Way We Age? The Gerontologist 2009.
37. Ruiz-Parraga GT, Lopez-Martinez AE, Gomez-Perez L. Factor structure and psychometric properties of the resilience scale in a spanish chronic musculoskeletal pain sample. The journal of pain : official journal of the American Pain Society 2012;13(11):1090-8.
38. Gonzalez SP, Detling N, Galli NA. Case studies of developing resilience in elite sport: Applying theory to guide interventions. Journal of Sport Psychology in Action 2016:1-12.
39. Elliott AM, Burton CD, Hannaford PC. Resilience does matter: evidence from a 10-year cohort record linkage study. BMJ Open 2014;4(1).
40. Ericsson KA. Attaining excellence through deliberate practice: Insights from the study of expert performance. Teaching and learning: The essential readings 2002:4-37.
41. Forgeard M. J. C. J, E., Kern, M. L., Seligman, M. E. P. Doing the right thing: Measuring wellbeing for public policy. International Journal of Wellbeing 2011;1(1):79-106.



For peer review only - http://bmjopen.bmj.com/site/about/guidelines.xhtml

**Table 1** Participant characteristics

| Alias | Age range[1] | BMI | Joint pain | OA | Past injury | Arthroscopic joint surgery | Joint replacement | Years post retirement[1] | UK professional seasons[1] | Considering the benefits and risks of cricket I would do the same again | I would recommend cricket to significant others |
|---|---|---|---|---|---|---|---|---|---|---|---|
| Cam | 51 to 55 | Overweight | Y | N | Y | N | N | 26 to 30 | 1 to 5 | strongly agree | strongly agree |
| Dan | 56 to 60 | Normal | Y | Y | Y | Y | N | 26 to 30 | 6 to 10 | strongly agree | strongly agree |
| Dom | 61 to 65 | Obese | N | Y | N | Y | Y | 26 to 30 | 16 to 20 | strongly agree | strongly agree |
| Gus | 56 to 60 | Overweight | N | N | N | N | N | 11 to 15 | 1 to 5 | strongly agree | strongly agree |
| Joe | 61 to 65 | Overweight | N | N | Y | N | N | 31 to 35 | 16 to 20 | agree | agree |
| Leo | 76 to 80 | Normal | Y | Y | Y | Y | Y | 36 to 40 | 1 to 5 | strongly agree | strongly agree |
| Ned | 56 to 60 | Overweight | Y | N | N | N | N | 16 to 20 | 16 to 20 | strongly agree | strongly agree |
| Tim | 36 to 40 | Overweight | Y | Y | Y | Y | N | 6 to 10 | NR | strongly agree | strongly agree |
| Wes | 66 to 70 | Overweight | Y | N | Y | N | N | 26 to 30 | 21 to 25 | strongly agree | strongly agree |
| Ben | 56 to 60 | Overweight | Y | Y | Y | Y | Y | 21 to 25 | 11 to 15 | strongly agree | strongly agree |
| Fin | 31 to 35 | Overweight | Y | N | Y | Y | N | 6 to 10 | 6 to 10 | strongly agree | agree |
| Guy | 46 to 50 | Obese | Y | N | Y | Y | N | 21 to 25 | 1 to 5 | strongly agree | strongly agree |
| Jim | 66 to 70 | Overweight | Y | Y | Y | Y | Y | 21 to 25 | 21 to 25 | strongly agree | strongly agree |
| Ken | 56 to 60 | Overweight | Y | Y | Y | N | N | 26 to 30 | 6 to 10 | strongly agree | strongly agree |
| Lee | 46 to 50 | Overweight | Y | N | N | Y | N | 11 to 15 | 6 to 10 | strongly agree | strongly agree |
| Ric | 66 to 70 | Obese | Y | Y | Y | N | Y | 16 to 20 | 1 to 5 | agree | agree |
| Ron | 51 to 55 | Normal | Y | Y | Y | Y | N | 16 to 20 | 16 to 20 | undecided | undecided |
| Sam | 56 to 60 | Overweight | Y | Y | Y | Y | N | 21 to 25 | 16 to 20 | strongly agree | strongly agree |

Participants above the horizontal line strongly agreed or agreed that participation in cricket resulted in an increase in current physical activity level, participants below the horizontal line strongly agreed or agreed that participation in cricket resulted in a decrease in current physical activity levels; [1] Ranges were reported rather than absolute values to assure participants' anonymity; Y = yes; N = no; NR = Not reported; BMI (body mass index) = categorised with reference to WHO international classification guidelines (normal weight: 18.9–24.9 kg/m2, overweight: 25.0–29.9 kg/m2, obese: ≥30.0 kg/m2)[20]; Joint pain = 'Do you experience pain, discomfort, or have a problem with your: hip(s) or groin, knee(s), ankle(s), spine (back or neck), shoulder(s), elbow(s), wrist(s), finger(s) or hand(s)'; OA (osteoarthritis) = 'Have you ever been told you have wear and tear, degeneration or osteoarthritis by a doctor?'; Past injury = 'Have you ever had any injuries leading to more than 4 weeks of reduced participation in exercise, training or sport?'; Arthroscopic joint surgery = one or more arthroscopic joint surgery; Joint replacement = have you ever had joint replacement surgery?; UK professional seasons = number of seasons playing professional cricket in the UK; Considering the benefits and risks of cricket I would do the same again = Considering the benefits and risks of my previous



For peer review only - http://bmjopen.bmj.com/site/about/guidelines.xhtml

participation in cricket, I would do the same again (responses on a 5 point Likert-scale: strongly agree, agree, undecided, disagree, strongly disagree); I would recommend cricket to significant others = Considering the benefits and risks of my previous participation in cricket, I would recommend this to my children, relatives or close friends (responses on a 5 point Likert-scale: strongly agree, agree, undecided, disagree, strongly disagree)

For peer review only



For peer review only - http://bmjopen.bmj.com/site/about/guidelines.xhtml

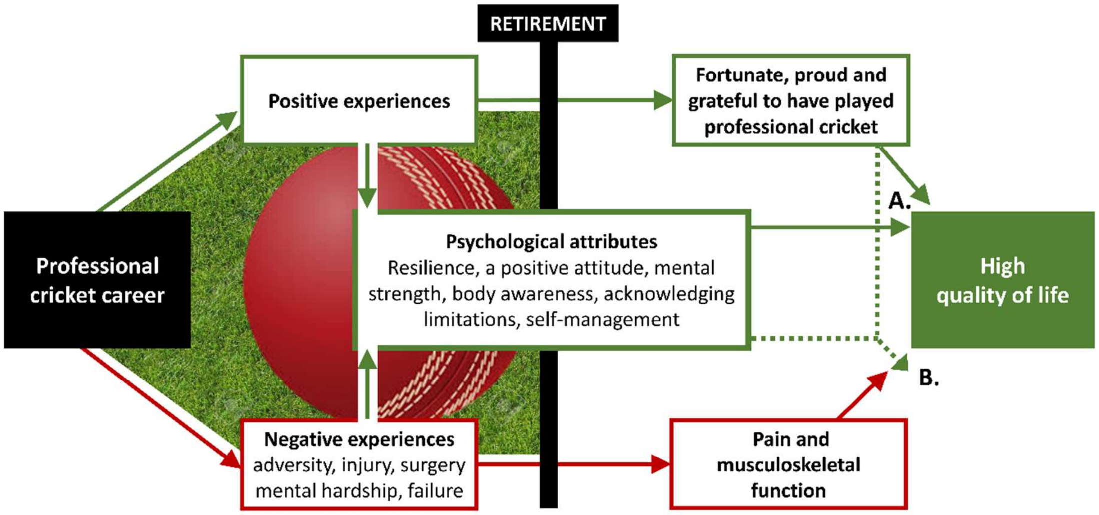


**Figure 1** A conceptual flow diagram summarising main findings and potential interactions between key themes

Green arrows and boxes represent factors with potential to *positively* impact quality of life;
Red arrows and boxes represent factors with potential to *negatively* impact quality of life;
A. Reflecting positively upon past experience and psychological attributes may positively impact quality of life following retirement from professional cricket;
B. Reflecting positively upon past experience and psychological attributes may reduce the potential for pain, osteoarthritis and physical limitations to negatively impact quality of life after retirement from professional cricket;
Together, A. and B. provide one potential explanation for high quality of life despite a high prevelance of pain, osteoarthirits and physical limitations in this sample of retired professional cricketers

For peer review only



For peer review only - http://bmjopen.bmj.com/site/about/guidelines.xhtml

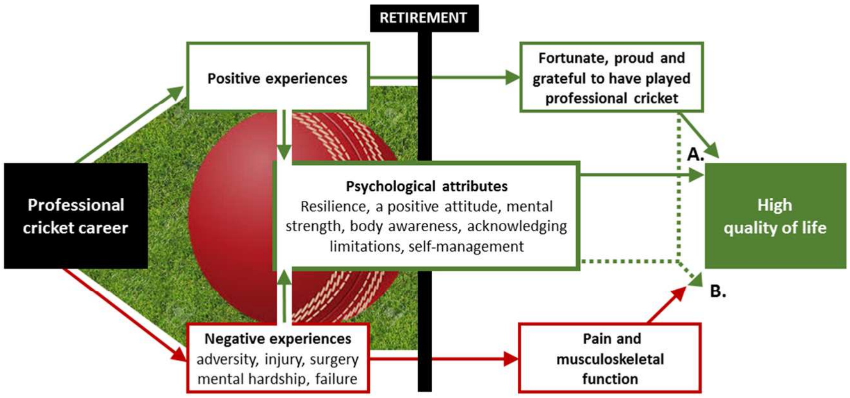


Figure 1 A conceptual flow diagram summarising main findings and potential interactions between key themes

Green arrows and boxes represent factors with potential to positively impact quality of life;
Red arrows and boxes represent factors with potential to negatively impact quality of life;
A. Reflecting positively upon past experience and psychological attributes may positively impact quality of life following retirement from professional cricket;
B. Reflecting positively upon past experience and psychological attributes may reduce the potential for pain, osteoarthritis and physical limitations to negatively impact quality of life after retirement from professional cricket;
Together, A. and B. provide one potential explanation for high quality of life despite a high prevelance of pain, osteoarthirits and physical limitations in this sample of retired professional cricketers

338x190mm (96 x 96 DPI)

only

**Supplementary Appendix 1** Semi-structured interview guide

| |
|---|
| 1. Can you describe any physical activity, exercise or sport that you currently take part in? |
| 2. Has that remained fairly constant since you retired from cricket or has it changed over the years? |
| 3. Have you played cricket again since retiring? Why/why not? |
| 4. What was your motivation for playing cricket? |
| 5. Are you as physically active as you would like to be? If no, why not? How does this make you feel? |
| 6. What is your motivation for taking part in physical activity/exercise/sport? |
| 7. How important is being physically active to you? (If important, why is it important? / If not important, has it always been this way?) |
| 8. Does the type of physical activity that you do matter to you, or would you be satisfied taking part in any form of physical activity?<br>(ask about specific forms of exercise that they find *dissatisfying* and why) |
| 9. What physical activity goals are you currently trying to achieve, if any? |
| 10. What are the barriers or challenges, if any, that impact on your ability to be physically active? |
| 11. Do you think that retired cricketers face the same challenges with being physically active as the general population, or are they unique or different in some way? |
| 12. Some retired cricketers become physically inactive, what advice would you give to help them maintain a physically active lifestyle after retiring from cricket? |
| 13. If you wanted to increase your physical activity levels, what do you think would help you to do so? |
| 14. Can you describe any positive or negative impacts that your previous participation in cricket has had upon your current physical activity patterns? |
| 15. If you hadn't played professional cricket, do you think that you would be more or less active, than you currently are? |
| 16. Does your current ability to participate in physical activity impact upon your quality of life? If yes, in what ways? If no, why not? |
| 17. Overall how satisfied are you with your current quality of life?<br>Do you think that this is related to your past career in cricket? |
| 18. Is there anything more you would like to add about your experiences with physical activity after retiring from professional cricket? |

BMJ Open

# Quality of life following retirement from professional cricket: retired players' perspectives

| | |
|---|---|
| Journal: | *BMJ Open* |
| Manuscript ID | bmjopen-2017-016541.R1 |
| Article Type: | Research |
| Date Submitted by the Author: | 12-Apr-2017 |
| Complete List of Authors: | Filbay, Stephanie; University of Oxford, Arthritis Research UK Centre for Sport, Exercise & Osteoarthritis, Nuffield Department of Orthopaedics, Rheumatology & Musculoskeletal Sciences<br>Bishop, Felicity; University of Southampton, Psychology<br>Peirce, Nicholas; Loughborough University, National Centre for Sport and Exercise Medicine & National Cricket Performance Centre<br>Jones, Mary; University of Oxford, Arthritis Research UK Centre for Sport, Exercise & Osteoarthritis, Nuffield Department of Orthopaedics, Rheumatology & Musculoskeletal Sciences<br>Arden, Nigel; University of Oxford, Arthritis Research UK Centre for Sport, Exercise & Osteoarthritis, Nuffield Department of Orthopaedics, Rheumatology & Musculoskeletal Sciences |
| <b>Primary Subject Heading</b>: | Sports and exercise medicine |
| Secondary Subject Heading: | Qualitative research |
| Keywords: | SPORTS MEDICINE, Osteoarthritis, PAIN MANAGEMENT, QUALITATIVE RESEARCH, Resilience |
| | |

SCHOLARONE™
Manuscripts

# Quality of life following retirement from professional cricket: retired players' perspectives

**Filbay S.R, Bishop F.L, Peirce N, Jones M.E, Arden N.K.**

Filbay S.R.

Arthritis Research UK Centre for Sport, Exercise & Osteoarthritis, Nuffield Department of Orthopaedics, Rheumatology & Musculoskeletal Sciences, University of Oxford
Botnar Research Centre, Windmill Rd, Oxford UK OX3 7LD
t: 07 414 064 683 | e: stephanie.filbay@uq.net.au | twitter: @stephfilbay

Bishop F.L.

Psychology department, University of Southampton, Southampton, UK. SO17 1BJ
t: +44 (0)23 8059 9020 | e: F.L.Bishop@southampton.ac.uk

Peirce N.

National Centre for Sport and Exercise Medicine & National Cricket Performance Centre, Loughborough University, Leicester, LE11 3TU UK
t: + 44 (0) 1509 228663 | e: nick.peirce@ecb.co.uk

Jones M.E.

Nuffield Department of Orthopaedics, Rheumatology & Musculoskeletal Sciences, University of Oxford
Botnar Research Centre, Windmill Rd, Oxford UK OX3 7LD
t: 01865 223436 | e: mary.jones@oriel.ox.ac.uk

Arden N.K.

Arthritis Research UK Centre for Sport, Exercise & Osteoarthritis, Nuffield Department of Orthopaedics, Rheumatology & Musculoskeletal Sciences, University of Oxford
Botnar Research Centre, Windmill Rd, Oxford UK OX3 7LD
t: +44 (0)1865 737859 | e: nigel.arden@ndorms.ox.ac.uk

For peer review only

**Word count** 3984 (4000 Word Limit)


## ABSTRACT

### Objectives

Retired professional cricketers shared unique experiences and may possess specific psychological attributes with potential to influence quality of life (QOL). Additionally, pain and osteoarthritis can be common in retired athletes which may negatively impact QOL. However, QOL in retired athletes is poorly understood.

This study explores the following questions from the personal perspective of retired-cricketers: How do retired cricketers perceive and experience musculoskeletal pain and function in daily life? Are there any psychological attributes that might enhance or hinder retired cricketers' QOL?

### Design

A qualitative study using semi-structured interviews, which were subject to inductive, thematic analysis. A data-driven, iterative approach to data coding was employed.

### Setting

All participants had lived and played professional cricket in the United Kingdom and were living in the UK or abroad at the time of interview.

### Participants


For peer review only

18 male participants, aged a mean 57±11 (range 34 to 77) years had played professional cricket for a mean 12±7 seasons and had been retired from professional cricket on average 23±9 years.

## Results

Fifteen participants reported pain or joint difficulties and all but one was satisfied with their QOL. Most retired cricketers reflected upon experiences during their cricket career that may be associated with the psychological attributes that these individuals shared, including resilience and a positive attitude. Additional attributes included a high sense of body awareness, an ability to self-manage pain and adapt lifestyle choices to accommodate physical limitations. Participants felt fortunate and proud to have played professional cricket, which may have further contributed to the high QOL in this group of retired cricketers.

## Conclusions

Most retired cricketers in this study were living with pain or joint difficulties. Despite this, all but one was satisfied or very satisfied with their QOL. This may be partly explained by the positive psychological attributes that these retired cricketers shared.

## Strengths and limitations of this study

- A strength of this study was the strong themes and consistent patterns that emerged through the interviews and the deep reflection and open, personal experiences that retired-cricketers shared that enriched the findings of this study.
- Since participants had been retired from professional cricket for an average of 23 years, we gained unique insight into longer-term quality of life after professional sport which had not been previously explored.

For peer review only

- It is not known whether cricket participation contributed to the development of positive psychological attributes or whether these largely pre-existed prior to cricket participation or developed after retirement.
- Limitations include generalisability and the possibility of selection bias, whereby retired cricketers with specific characteristics may be more likely to desire participation in a qualitative interview study.

## Funding

Dr Filbay was awarded a research fellowship from the Arthritis Research UK Centre for Sport, Exercise and Osteoarthritis to support this research. Dr Arden and Mary Jones have received an unrestricted research grant from the England and Wales Cricket Board. The parent cross-sectional study from which participants were recruited received funding from the Arthritis Research UK Centre for Sport, Exercise and Osteoarthritis, as well as the England and Wales Cricket Board.

## Competing interests

Dr Arden and Mary Jones have received an unrestricted research grant from the England and Wales Cricket Board. Dr Peirce is employed as the Chief Medical Officer of the England and Wales Cricket Board. Dr Filbay and Dr Bishop have nothing to disclose.

For peer review only

## Introduction

Research surrounding the long-term outcomes of a professional sporting career has largely focused on negative impacts rather than investigating the potential for positive psychological impacts that persist beyond retirement.[1] On retirement, athletes commonly experience negative emotions and reduced life satisfaction while transitioning and adapting to an alternative lifestyle,[2] but very little is known about life after this transition. After retirement, athletes are at increased risk of developing osteoarthritis, predominately due to a history of sport-related injury, and this can result in pain and activity limitations.[3-7] However, retired athletes may possess psychological attributes that could enhance quality of life and facilitate effective coping with pain and musculoskeletal impairment. Additionally, retired athletes may feel proud and accomplished to have played professional sport which could have positive impacts on QOL. On the other hand, functional limitations in individuals with high athletic identity could negatively impact QOL if their physical activity desires are not satisfied. These complex possible influences on the QOL of retired athletes are poorly understood.

Professional cricket is all-encompassing, the duration of play exceeds most other sports with formats including entire day matches and test matches lasting up to five consecutive days. In cricket, different playing positions have distinct psychological demands. A batsman facing a ball is under great pressure to perform, one slip of concentration or an incorrect decision can see him out of the game with no further opportunity to contribute positively to the team. A fielder in a slip position maintains prolonged intense focus in preparation for a potential catch with an outcome of success or failure determined by a momentary reaction. The demands of cricket contrast most other team sports where players have greater opportunity to correct

For peer review only

performance and ongoing involvement within a game. Thus, professional cricketers face unique competitive demands that require advanced psychological and behavioural control to manage stress and perform under pressure.[8 9] The ability to deal with such stressors will in part, determine the success of a professional cricketer. Psychological attributes associated with a successful cricket career include resilience, mental toughness, self-belief, optimism, coping with adversity and confidence.[10-12] Although these psychological attributes have been observed in professional cricketers, it is not known whether they persist after retirement. The psychological attributes that retired cricketers possess have potential to benefit other aspects of life and reduce the impact of musculoskeletal pain following retirement from cricket.

We undertook a qualitative study to provide an in-depth analysis of life after retirement from professional cricket, from the personal perspective of retired-cricketers. The a priori aim of this qualitative study was to explore physical activity after retirement from professional cricket. However, the study also captured participants' broader perspectives regarding QOL and prominent themes linking QOL to professional cricket emerged during the initial inductive analysis. This paper focuses on these emergent themes, which were explored while considering two sensitising questions: How do retired cricketers perceive and experience musculoskeletal pain and function in daily life? Are there any psychological attributes that might enhance or hinder retired cricketers' QOL? A complementary manuscript will address physical activity after retirement from professional cricket.

## Methods

### *Ethical considerations*

This study has been approved by Medical Sciences Inter-divisional Research Ethics Committee (IDREC), University of Oxford (reference number R45197/RE001).

***Participants and recruitment***

Participants were purposively sampled from a larger cohort study of 187 retired professional English cricketers who recently completed a cross-sectional questionnaire collecting information on demographics, playing history, current pain, medical history (including doctor diagnosed osteoarthritis, arthroscopic surgery and joint replacement surgery) and injury history (Jones et al. *under review*). In order to facilitate a balanced exploration of physical activity behaviours, barriers, and facilitators, we purposively sampled individuals based on their responses to two questions from the cross-sectional study assessing perceived impact of previous cricket participation upon current physical activity levels: i) Strongly agree or agree that participation in cricket has resulted in an *increase* in current physical activity level (n=46, 42%), or ii) Strongly agree or agree that participation in cricket has resulted in a *decrease* in current physical activity levels (n= 27, 25%). We aimed to include an equal number of participants from each of these groups.

Only individuals from the larger cross-sectional study who indicated a willingness to participate in future sport-related research were invited (by email) into the current study. Among those expressing an interest in this study, participants were then purposively sampled to ensure men of a range of ages were interviewed. Interviews were conducted until the point of data saturation, defined a priori as the point at which no new themes were identified from four consecutive interviews (two from participants with reduced physical activity and two from participants with increased physical activity). After which, additional interviews were performed as deemed appropriate to explore ideas and themes in greater detail after following

For peer review only

the semi-structured interview guide. These final interviews were also used to affirm data saturation. If new themes emerged from these final interviews, additional interviews would be conducted until data saturation is satisfied.

A total of 42 email invitations were sent to eligible participants, 19 emails received no response, 2 individuals declined to participate, 2 individuals were unavailable due to overseas travel and 1 individual indicated a willingness to participate but did not respond to further correspondence. Data saturation was achieved by the 14th interview. An additional four interviews were carried out after data saturation and no new themes emerged. Data from all 18 interviews were used for analysis.

***Interviews***

Audio recorded semi-structured telephone interviews were performed by S.R.F. Informed verbal consent was obtained from each participant prior to taking part. Interviews were transcribed verbatim by a research assistant. All transcripts were de-identified during transcription, and an alias was assigned to each participant. The 18 interviews were on average 26 minutes in length (ranging from 18 to 37 minutes).

A semi-structured interview guide was used to assure that key areas of interest were covered while enabling the researcher to adapt the interview guide to elicit relevant and rich information from respondents by probing and prompting for additional details.[13] The interview guide contained open-ended questions to provide participants with the greatest opportunity to consider personal perspectives and experiences (Appendix 1). The interview

For peer review only

guide was iteratively adapted throughout the interviews to incorporate any additional issues of importance to respondents (for example, by adding a question to explore their relationship with cricket post-retirement). Interviewees were given the opportunity to contribute any additional information at the end of the interview.

***Analysis procedure***

An inductive thematic approach was used[14 15] facilitated by NVivo version 11 software.[16] Following each interview, the interviewer summarised and reflected upon initial ideas in a study journal. Transcripts were read and re-read with accompanying audio to identify all information relevant to the research aims.[17] An iterative approach to data coding was employed. All data that might be relevant to the aims of the study were coded into multiple categories to be refined and analysed for themes in subsequent stages of analysis.[14] During the earlier stages of analysis, no attention was given to the prevalence of themes and coding was performed diversely and inclusively, withholding further interpretation. In line with an inductive thematic approach, coding was data-driven, performed without reference to a pre-existing coding structure and with little engagement with literature to avoid sensitization to specific themes.[14 17]

During the later stages of analysis, the data was further analysed for repeated patterns, codes were sorted into a hierarchical structure representing themes and subthemes, overlapping themes were merged, and those outside the scope of the current study were filed separately. These themes and sub-themes were repeatedly reviewed and refined to confirm external heterogeneity and internal homogeneity within themes and to ensure an accurate representation of the entire dataset.[14 18] During this stage, all transcripts were re-read and the

For peer review only

study journal was revisited to check that themes accurately reflected the key issues discussed by participants. A selection of six transcripts representing participants with diverse physical activity patterns were analysed by a second investigator (F.L.B) blinded to the coding structure developed by the first author (S.R.F). A meeting was then held to discuss key themes, and a high level of agreement was achieved between investigators. Key themes and sub-themes will be described with reference to participant quotes.[14 18]

Following qualitative analysis, responses to relevant questions collected as part of the cross-sectional study were used to describe the study sample (including osteoarthritis status, age, previous surgery and length of professional cricket career). Questionnaire data was cross-checked for accuracy with participants' narratives and if disparities were present (e.g. a participant reported no current joint pain on questionnaire but described experiencing current joint pain during the interview), participant narratives were considered more reliable.

## Results

### *Participant characteristics*

The 18 participants who took part in the interviews were all male, aged a mean 57±11 (range 34 to 77) years, played professional cricket in the United Kingdom for a mean 12±7 (range 2 to 25) seasons and had been retired from professional cricket for an average 23±9 (range 7 to 38) years. The majority were overweight by World Health Organisation (WHO) criteria[19] (n=12, 67%) and three participants (17%) were classified as obese. Current joint pain or difficulties were common (n=15, 83%), 10 people (56%) had been told by a doctor that they had osteoarthritis, 12 participants (67%) had received at least one joint surgery and 5 participants (28%) had received one or more total joint replacement. More than two thirds (n=14, 78%) reported having ever had an injury that resulted in more than one month of

For peer review only

reduced participation in exercise, training or sport. Full participant characteristics are reported in Table 1.

[Insert Table 1]

***Pain and musculoskeletal function***

Fifteen retired cricketers described experiencing current pain and limitations with musculoskeletal function. For most individuals, this prevented them for taking part in specific activities, like running and recreational sports. Although this was described as frustrating by some, participants had made lifestyle modifications to accommodate these impairments and very few described a negative impact upon QOL.

> *Jim: 'I've had 13 operations over the years because of cricket injuries and resulting stuff from that and I'm just restricted in what I can do really. ..it doesn't make me feel, nothing really, as long as I can do the little bits I can I'm quite happy.'*

> *Sam: 'So there are little bits that frustrate me but I mean, I'm nearly 60 so you know, your body does get worn out if you do something as extreme as play a professional sport for 17 years, so yeah they're just things that exist, but no, I'm not that bothered.'*

***The ups and downs of professional cricket***

Retired-cricketers described experiencing mental hardship and psychological challenges during their cricket career. These included coming to terms with experiencing more failure than success and difficulty coping with pressure to perform. Almost all participants believed these experiences benefited other areas of life after retirement. Specifically, they described how attributes required to succeed on the cricket field, including mental toughness, confidence, and resilience, equipped them with tools to overcome post-retirement life

For peer review only

challenges, including managing chronic pain and establishing a successful career outside of cricket.

> *Lee 'It was just a constant battle, it was soul destroying at times but out of all that you know; hard work, disappointment, you know, being beaten down by people at times… I think it's given me the mental capacity just to, just get your head down and battle on. I think that's the main thing, you can have bad times in whatever you're doing, and you're going to feel really low, but I think just that mental capacity that you've learnt from cricket, from being a professional sportsmen, to just get back up on your feet and fight another day sort of thing is being part of where I've got to now, you know thanks to that basically.'*

Only one individual felt that the mental aspects of professional cricket had a negative impact upon life after retirement. Dan attributed difficulty coping with adversity and reduced confidence to having played professional cricket.

> *Dan: 'I wouldn't say I carry mental scars, but I think it's affected my personality.. ..probably I am less confident having been a professional sportsman than I would have been otherwise, because I think I have been beaten up quite a few times over the years, you know mentally, and it's taken its toll a little bit.'*

***Psychological attributes that may enhance quality of life***

*Resilience and a positive attitude*

Attributes that appeared to enhance QOL in this sample of retired cricketers, included resilience and a positive attitude. Almost all retired-cricketers in this study were resilient and had a positive attitude regarding pain and musculoskeletal impairment.

> *Dom: 'I would classify myself to be very, very lucky, probably very, very lucky to come out the other side, with just, at the moment, hip injuries that have been cured by hip operations… it's all been extremely positive, from a life style I've reached, yeah.'*

> *Ron: 'Keep a strong mind and you will have aliments depending on how much sport you have played and you just have to get on with it.''*

*Body awareness, acknowledging limitations and self-management*

For peer review only

There were other common attributes amongst the retired cricketers that appeared to have a positive impact upon QOL, these included a heightened sense of body awareness and confidence in self-managing physical ailments. Most participants had made positive adaptations and were mindful of their body's capabilities. Participants adhered to self-imposed activity limits and modified their activity choices on a day to day basis to avoid exacerbation of pain or an acute deterioration in physical function.

*Fin: '.. you also have a greater and wiser knowledge of what you should, could, and can and can't do. So from a positive point of view you know what you can and can't do and you know what's good for you and your body.'*

*Gus: 'I like to think that I know what my, what my boundaries are and I can just push them a little bit or to the limit that I think that, that my age will allow me to do... I definitely think that playing cricket made me certainly more aware and being involved in cricket for the amount of time that I had, I certainly have, have more knowledge of what I can and can't do.'*

*Fortunate, proud and grateful to have played professional cricket*

Reflecting upon their past career in cricket, all participants felt fortunate, proud or grateful to have had the opportunity to play professional cricket. All but one individual believed that overall, the positive effects of cricket outweighed the negative, and expressed no regret over playing professional cricket.

*Fin: 'No regrets what so ever. It was a great life. I had a great time. There were things that, when you come out of that sporting environment into the real world, if you like, and you realise that then its only things that other people dream of doing, is what you've done. So absolutely no regrets what so ever.'*

*Sam: 'You just play the hand your dealt and I never think there's any point having any regrets or bearing any grudges, it is what it is and you know I've had a great life out of it. ...I've made plenty of rubbish decisions, but I wouldn't change that fact, because all that failure and all that success has made me who I am and I'm quite happy being me now.'*

***Quality of life***

For peer review only

Irrespective of pain and joint difficulties, most retired-cricketers were very satisfied (n=11, 61%) or satisfied (n=6, 33%) with their QOL. No participants expressed that their QOL was impaired due to pain or physical issues. The only individual dissatisfied with his QOL attributed this entirely to current family difficulties.

Interviewer: Overall how satisfied are you with your current quality of life?

*Wes: '110%, cannot believe my luck. ..I am spoilt rotten, but I mean there's no point in being in a brilliant position, which I am, if you don't appreciate it, I mean I can see lots of people who don't know how lucky they are, well, I am glad to say I do.*

*Sam: 'I am absolutely delighted with it, I love it. I'm in a really good place mentally… things couldn't be better.'*

*Leo: 'Oh brilliant, I'm just very, very lucky, absolutely brilliant.'*

*Ken: 'I am very happy with it actually, and have been for a number of years.'*

*Joe: 'Good, yeah I'm very happy.'*

*Guy: 'Oh on a scale, I would say 8/9 out of 10; I think I am very satisfied.'*

*Gus: 'Well I am very happy, very happy and pleased with my quality of life.'*

*Dom: 'Very happy. ..very happy with where I am physically and mentally in what I do and what I want to do, yes.'*

*Ned: 'Oh generally, you know, very happy really, I think you know keeping things in perspective of, you know of how I am, my age, you know, what I've got, you know I can't complain really. I am really pretty pleased with the way things are.'*

***Summary of key themes***

Figure 1 provides a conceptual flow diagram summarising the main findings and providing an overview of how the key themes may interact to make a positive contribution to QOL after retirement.

[Insert Figure 1 here]

For peer review only

## Discussion

Current pain and physical limitations were common in this group of retired professional cricketers. Despite this, almost all retired cricketers were satisfied or very satisfied with their QOL. One potential explanation for this counterintuitive finding is the common psychological attributes that we found these individuals to possess, including resilience, a positive attitude, heightened body awareness, acknowledgement of physical limitations, and an ability to modify activity choices to effectively cope with fluctuations in pain and musculoskeletal function. Reflecting upon their past cricket career, participants felt very grateful and fortunate to have played professional cricket, and all but one felt the positive impacts of a career in cricket outweighed any negatives. This feeling of pride and accomplishment has further potential to positively influence QOL in this group of retired cricketers, irrespective of pain and physical impairment.

***Quality of life and flourishing***

We found that all but one retired-cricketer was satisfied with his QOL. Flourishing is a concept that represents an optimal QOL where an individual excels emotionally, psychologically and socially. In 2013, 20% of a representative sample of the UK general population were found to be 'flourishing' (possessing 9 of 10 signs of positive wellbeing including resilience, optimism, competence, self-esteem and meaning).[20] Retired cricketers who took part in the interviews exhibited many attributes consistent with the definition of flourishing. It is possible that a greater number of retired-cricketers 'flourish' in later life compared with the general population despite a higher prevalence of pain and osteoarthritis, although further research is necessary to explore this possibility.

For peer review only

Retired professional footballers with osteoarthritis reported worse health-related QOL compared with retired footballers without osteoarthritis.[21] However, most conventional QOL outcome measures (including the EQ-5D used in the study of retired footballers[21]) are influenced by pain and physical impairment, where the presence of pain and physical disability will result in a reduced QOL score, irrespective of the impact of pain or disability upon the individual.[22] Evaluation of the psychometric properties of QOL measures in retired sporting populations is warranted. The Flourishing Scale[23] or a QOL measure not driven by pain and function (such as the WHO-QOL[24]) may be preferable for use in retired sporting populations.

***Resilience and associated positive psychological attributes***

Resilience refers to responding to adversity with positive adaptation, and in doing so, restoring an internal sense of emotional and psychological balance.[25 26] Retired-cricketers in the present study shared resilient attributes, this was highlighted by their ability to maintain a positive attitude and adapt activity choices to effectively cope with fluctuations in pain and physical function. Prior research has found that athletes possess greater resilience and optimism than non-athletes[27] and successful cricketers are likely to be more resilient than less successful cricketers.[10 12] A high level of resilience is associated with increased QOL in a wide variety of disease-specific and chronic pain populations.[28-35] Resilient individuals also demonstrate more adaptive coping styles and better musculoskeletal pain adjustment.[36 37] Considering sporting injury is a risk factor for osteoarthritis, interventions to build resilience within a sporting setting[38] may have benefits that extend beyond success on the sporting field, with potential to enhance QOL in later life.

Despite these benefits, who is likely to become resilient and why is poorly understood.[39] The relationship between resilience and professional cricket is likely multifaceted. Exposure to the demands of cricket from a young age, including repetitive failure, disappointment, stress and adversity may facilitate the development of resilience. On the other hand, resilience may be influenced by a range of non-cricket related factors, and resilient individuals may be most likely to reach professional status resulting in a high level of resilience in professional cricketers. It is also possible that adversities faced during transition from sport and after retirement contributed to the high level of resilience in this group of retired cricketers.

***Cricket reflection***

Accomplishment refers to success, achievement or mastery at the highest possible level[40] and has been recognised as an important component of wellbeing.[41] Considering all retired-cricketers in this study were fortunate, proud or grateful to have played professional cricket it is possible that this accomplishment further contributed to the high QOL described by these individuals. A retired athlete's sense of accomplishment and fulfilment through past participation in sport is too often overlooked in research, despite potential to positively influence QOL.

***Study strengths and potential limitations***

It is not known whether cricket participation contributed to the development of positive psychological attributes or whether these largely pre-existed prior to cricket participation or developed after retirement. Prospective longitudinal studies would be required to answer this

For peer review only

question. Although participants were asked about their degree of satisfaction with their overall QOL, we did not explore personal beliefs about the definition of QOL. Consequently the question pertaining to QOL could have meant different things to different participants. Due to purposive recruitment, individuals reporting uncertainty regarding the impact of cricket upon their physical activity level were not invited into the study, reducing the generalisability of results. It is also possible that the study was subjected to selection bias, whereby retired cricketers with specific characteristics were more likely to desire participation in a qualitative interview study. A strength of this study was the strong themes and consistent patterns that emerged through the interviews resulting in saturation of data at 14 interviews, confirmed by the final four interviews that enabled further complimentary exploration into ideas and important themes. The interviewer was a physiotherapist with experience in building rapport with individuals and capturing issues of importance to an individual. Rapport was further strengthened by the interviewer's familiarity with cricket as a physiotherapist and cricket participant. These factors facilitated deep reflection and open, personal insights from retired-cricketers that enriched the findings of this study.

## Conclusion

Irrespective of chronic joint pain and physical limitations, all but one retired-cricketer in this study was satisfied or very satisfied with their QOL. Retired-cricketers shared common psychological attributes including resilience and a positive attitude that may partly explain the small impact pain and physical limitations appeared to have upon the QOL of these retired cricketers. Additionally, most retired cricketers had a high sense of body awareness and executed self-control to adapt activity choices and accept physical limitations. Participants felt fortunate and grateful to have played professional cricket and expressed that

For peer review only

the benefits of cricket participation outweigh any negative impacts. This study highlights fruitful areas for future research including i) flourishing in retired athletes despite pain and functional limitations, ii) the potential for positive psychological attributes to develop through sport participation and impact QOL after retirement, and iii) the effect of past accomplishment in sport on QOL across the lifespan. A better understanding of the relationship between sport participation and QOL could assist in promoting physical activity and facilitate strategies to enhance the positive impact of sport upon QOL.

## Acknowledgments

We would like to thank Angus Porter and the Professional Cricketers' Association (PCA) for assisting with recruitment and questionnaire development for the larger cross-sectional study from which study participants were purposively recruited.

## Author contributions

SRF, FLB, NP, NKA conceived and designed this qualitative study. SRF, MEJ recruited participants and extracted data form the cross-sectional cohort. SRF performed all interviews. SRF, FLB participated in the analysis. SRF drafted the first version of the manuscript. All authors contributed in revising the manuscript and gave their final approval of the submitted version.

## Data sharing

For peer review only

To view interview transcripts or additional participant quotes, please contact the corresponding author.

For peer review only

## References


1. Knights S, Sherry E, Ruddock-Hudson M. Investigating Elite End-of-Athletic-Career Transition: A Systematic Review. *Journal of Applied Sport Psychology* 2016:1-18. doi: 10.1080/10413200.2015.1128992
2. Park S, Lavallee D, Tod D. Athletes' career transition out of sport: a systematic review. *International Review of Sport and Exercise Psychology* 2013;6(1):22-53. doi: 10.1080/1750984X.2012.687053
3. Spector T, Harris P, Hart DJ, et al. Risk of osteoarthritis associated with long-term weight-bearing sports. *Arthritis and rheumatism* 1996;39(6):988-95.
4. Thelin N, Holmberg S, Thelin A. Knee injuries account for the sports-related increased risk of knee osteoarthritis. *Scandinavian journal of medicine & science in sports* 2006;16(5):329-33.
5. Gelber AC, Hochberg MC, Mead LA, et al. Joint injury in young adults and risk for subsequent knee and hip osteoarthritis. *Annals of internal medicine* 2000;133(5):321-28.
6. Gouttebarge V, Inklaar H, Backx F, et al. Prevalence of osteoarthritis in former elite athletes: a systematic overview of the recent literature. *Rheumatology international* 2015;35(3):405-18. doi: 10.1007/s00296-014-3093-0 [published Online First: 2014/07/20]
7. Arliani GG, Astur DC, Yamada RKF, et al. Early osteoarthritis and reduced quality of life after retirement in former professional soccer players. *Clinics* 2014;69(9):589-94. doi: 10.6061/clinics/2014(09)03
8. Neil R, Bowles HCR, Fleming S, et al. The experience of competition stress and emotions in cricket. *Sport Psychologist* 2016;30(1):76-88. doi: 10.1123/tsp.2014-0077
9. Thelwell RC, Weston NJ, Greenlees IA. Batting on a sticky wicket: Identifying sources of stress and associated coping strategies for professional cricket batsmen. *Psychology of Sport and Exercise* 2007;8(2):219-32.
10. Bull SJ, Shambrook CJ, James W, et al. Towards an understanding of mental toughness in elite English cricketers. *Journal of Applied Sport Psychology* 2005;17(3):209-27. doi: 10.1080/10413200591010085
11. Weissensteiner J, Abernethy B, Farrow D. Towards the development of a conceptual model of expertise in cricket batting: A grounded theory approach. *Journal of Applied Sport Psychology* 2009;21(3):276-92. doi: 10.1080/10413200903018675
12. Weissensteiner JR, Abernethy B, Farrow D, et al. Distinguishing psychological characteristics of expert cricket batsmen. *Journal of Science and Medicine in Sport* 2012;15(1):74-79.
13. Jones I, Brown L, Holloway I. Qualitative Research in Sport and Physical Activity: SAGE Publications 2012.
14. Braun V, Clarke V. Using thematic analysis in psychology. *Qualitative Research in Psychology* 2006;3(2):77-101. doi: 10.1191/1478088706qp063oa
15. Thomas DR. A General Inductive Approach for Analyzing Qualitative Evaluation Data. *American Journal of Evaluation* 2006;27(2):237-46. doi: 10.1177/1098214005283748
16. Bazeley P, Jackson K. Qualitative data analysis with NVivo. Los Angeles: SAGE 2013.
17. Tuckett AG. Applying thematic analysis theory to practice: A researcher's experience. *Contemporary Nurse* 2005;19(1-2):75-87. doi: 10.5172/conu.19.1-2.75
18. Patton MQ. Qualitative evaluation and research methods1990.
19. Organisation WH. Obesity: Preventing and managing the global epidemic. Report of a WHO Consultation on Obesity, Geneva,. *WHO/NUT /NCD/98 1, Geneva, 1998* 3-5 June 1997
20. Huppert FA, So TTC. Flourishing Across Europe: Application of a New Conceptual Framework for Defining Well-Being. *Social Indicators Research* 2013;110(3):837-61. doi: 10.1007/s11205-011-9966-7


For peer review only

21. Turner AP, Barlow JH, Heathcote-Elliot C. Long term health impact of playing professional football in the United Kingdom. *British Journal of Sports Medicine* 2000;34(5):332-37. doi: 10.1136/bjsm.34.5.332
22. Filbay SR. Longer-term quality of life following anterior cruciate ligament injury and reconstruction. *PhD Thesis, School of Health and Rehabilitation Sciences, The University of Queensland* 2016;1st July 2016 doi: 10.14264/uql.2016.384
23. Diener E, Wirtz D, Tov W, et al. New Well-being Measures: Short Scales to Assess Flourishing and Positive and Negative Feelings. *Social Indicators Research* 2010;97(2):143-56. doi: 10.1007/s11205-009-9493-y
24. Group TW. Development of the World Health Organization WHOQOL-BREF Quality of Life Assessment. *Psychological medicine* 1998;28(3):551-58. doi: 10.1017/S0033291798006667
25. Galli N, Gonzalez SP. Psychological resilience in sport: A review of the literature and implications for research and practice. *International Journal of Sport and Exercise Psychology* 2015;13(3):243-57. doi: 10.1080/1612197X.2014.946947
26. Schinke RJ, Battochio RC, Dube TV, et al. Adaptation Processes Affecting Performance in Elite Sport. *Journal of Clinical Sport Psychology* 2012;6(2):180-95. doi: doi:10.1123/jcsp.6.2.180
27. Guillén F, Laborde S. Higher-order structure of mental toughness and the analysis of latent mean differences between athletes from 34 disciplines and non-athletes. *Personality and Individual Differences* 2014;60:30-35. doi: 10.1016/j.paid.2013.11.019
28. Li MY, Yang YL, Liu L, et al. Effects of social support, hope and resilience on quality of life among Chinese bladder cancer patients: A cross-sectional study. *Health and Quality of Life Outcomes* 2016;14(1) doi: 10.1186/s12955-016-0481-z
29. Ring A, Jacoby A, Baker GA, et al. Does the concept of resilience contribute to understanding good quality of life in the context of epilepsy? *Epilepsy and Behavior* 2016;56:153-64. doi: 10.1016/j.yebeh.2016.01.002
30. Wu WW, Tsai SY, Liang SY, et al. The Mediating Role of Resilience on Quality of Life and Cancer Symptom Distress in Adolescent Patients With Cancer. *Journal of Pediatric Oncology Nursing* 2015;32(5):304-13. doi: 10.1177/1043454214563758
31. Vitali SA. Finding quality of life despite MS: Harnessing resilience. *International MS Journal* 2011;17(3):94-99.
32. Frigiola A, Bull C, Wray J. Exercise capacity, quality of life, and resilience after repair of tetralogy of Fallot: A cross-sectional study of patients operated between 1964 and 2009. *Cardiology in the Young* 2014;24(1):79-86. doi: 10.1017/S1047951112002296
33. Manne SL, Myers-Virtue S, Kashy D, et al. Resilience, Positive Coping, and Quality of Life among Women Newly Diagnosed with Gynecological Cancers. *Cancer Nursing* 2015;38(5):375-82. doi: 10.1097/NCC.0000000000000215
34. Yazdi-Ravandi S, Taslimi Z, Saberi H, et al. The role of resilience and age on quality of life in patients with pain disorders. *Basic and Clinical Neuroscience* 2013;4(1):24-30. doi: 10.1001/archinte.163.20.2433;
35. Bergman S, Jacobsson LT, Herrstrom P, et al. Health status as measured by SF-36 reflects changes and predicts outcome in chronic musculoskeletal pain: a 3-year follow up study in the general population. *Pain* 2004;108(1-2):115-23. doi: 10.1016/j.pain.2003.12.013 [published Online First: 2004/04/28]
36. Hildon Z, Montgomery SM, Blane D, et al. Examining Resilience of Quality of Life in the Face of Health-Related and Psychosocial Adversity at Older Ages: What is "Right" About the Way We Age? *The Gerontologist* 2009 doi: 10.1093/geront/gnp067
37. Ruiz-Parraga GT, Lopez-Martinez AE, Gomez-Perez L. Factor structure and psychometric properties of the resilience scale in a spanish chronic musculoskeletal pain sample. *The journal of pain : official journal of the American Pain Society* 2012;13(11):1090-8. doi: 10.1016/j.jpain.2012.08.005 [published Online First: 2012/10/16]

38. Gonzalez SP, Detling N, Galli NA. Case studies of developing resilience in elite sport: Applying theory to guide interventions. *Journal of Sport Psychology in Action* 2016:1-12. doi: 10.1080/21520704.2016.1236050

39. Elliott AM, Burton CD, Hannaford PC. Resilience does matter: evidence from a 10-year cohort record linkage study. *BMJ Open* 2014;4(1) doi: 10.1136/bmjopen-2013-003917

40. Ericsson KA. Attaining excellence through deliberate practice: Insights from the study of expert performance. *Teaching and learning: The essential readings* 2002:4-37.

41. Forgeard M. J. C. J, E., Kern, M. L., Seligman, M. E. P. Doing the right thing: Measuring wellbeing for public policy. *International Journal of Wellbeing* 2011;1(1):79-106. doi: 10.5502/ijw.v1i1.15

For peer review only

**Table 1** Participant characteristics

| Alias | Age range[1] | BMI | Joint pain | OA | Past injury | Arthroscopic joint surgery | Joint replacement | Years post retirement[1] | UK professional seasons[1] | Considering the benefits and risks of cricket I would do the same again | I would recommend cricket to significant others |
|---|---|---|---|---|---|---|---|---|---|---|---|
| Cam | 51 to 55 | Overweight | Y | N | Y | N | N | 26 to 30 | 1 to 5 | strongly agree | strongly agree |
| Dan | 56 to 60 | Normal | Y | Y | Y | Y | N | 26 to 30 | 6 to 10 | strongly agree | strongly agree |
| Dom | 61 to 65 | Obese | N | Y | N | Y | Y | 26 to 30 | 16 to 20 | strongly agree | strongly agree |
| Gus | 56 to 60 | Overweight | N | N | N | N | N | 11 to 15 | 1 to 5 | strongly agree | strongly agree |
| Joe | 61 to 65 | Overweight | N | N | Y | N | N | 31 to 35 | 16 to 20 | agree | agree |
| Leo | 76 to 80 | Normal | Y | Y | Y | Y | Y | 36 to 40 | 1 to 5 | strongly agree | strongly agree |
| Ned | 56 to 60 | Overweight | Y | N | N | N | N | 16 to 20 | 16 to 20 | strongly agree | strongly agree |
| Tim | 36 to 40 | Overweight | Y | Y | Y | Y | N | 6 to 10 | NR | strongly agree | strongly agree |
| Wes | 66 to 70 | Overweight | Y | N | Y | N | N | 26 to 30 | 21 to 25 | strongly agree | strongly agree |
| Ben | 56 to 60 | Overweight | Y | Y | Y | Y | Y | 21 to 25 | 11 to 15 | strongly agree | strongly agree |
| Fin | 31 to 35 | Overweight | Y | N | Y | Y | N | 6 to 10 | 6 to 10 | strongly agree | agree |
| Guy | 46 to 50 | Obese | Y | N | Y | Y | N | 21 to 25 | 1 to 5 | strongly agree | strongly agree |
| Jim | 66 to 70 | Overweight | Y | Y | Y | Y | Y | 21 to 25 | 21 to 25 | strongly agree | strongly agree |
| Ken | 56 to 60 | Overweight | Y | Y | Y | N | N | 26 to 30 | 6 to 10 | strongly agree | strongly agree |
| Lee | 46 to 50 | Overweight | Y | N | N | Y | N | 11 to 15 | 6 to 10 | strongly agree | strongly agree |
| Ric | 66 to 70 | Obese | Y | Y | Y | N | Y | 16 to 20 | 1 to 5 | agree | agree |
| Ron | 51 to 55 | Normal | Y | Y | Y | Y | N | 16 to 20 | 16 to 20 | undecided | undecided |
| Sam | 56 to 60 | Overweight | Y | Y | Y | Y | N | 21 to 25 | 16 to 20 | strongly agree | strongly agree |

Participants above the horizontal line strongly agreed or agreed that participation in cricket resulted in an increase in current physical activity level, participants below the horizontal line strongly agreed or agreed that participation in cricket resulted in a decrease in current physical activity levels; [1] Ranges were reported rather than absolute values to assure participants' anonymity; Y = yes; N = no; NR = Not reported; BMI (body mass index) = categorised with reference to WHO international classification guidelines (normal weight: 18.9–24.9 kg/m2, overweight: 25.0–29.9 kg/m2, obese: ≥30.0 kg/m2)[19]; Joint pain = 'Do you experience pain, discomfort, or have a problem with your: hip(s) or groin, knee(s), ankle(s), spine (back or neck), shoulder(s), elbow(s), wrist(s), finger(s) or hand(s)'; OA (osteoarthritis) = 'Have you ever been told you have wear and tear, degeneration or osteoarthritis by a doctor?'; Past injury = 'Have you ever had any injuries leading to more than 4 weeks of reduced participation in exercise, training or sport?'; Arthroscopic joint surgery = one or more arthroscopic joint surgery; Joint replacement = have you ever had joint replacement surgery?; UK professional seasons = number of seasons playing professional cricket in the UK; Considering the benefits and risks of cricket I would do the same again = Considering the benefits and risks of my previous

For peer review only

participation in cricket, I would do the same again (responses on a 5 point Likert-scale: strongly agree, agree, undecided, disagree, strongly disagree); I would recommend cricket to significant others = Considering the benefits and risks of my previous participation in cricket, I would recommend this to my children, relatives or close friends (responses on a 5 point Likert-scale: strongly agree, agree, undecided, disagree, strongly disagree)

For peer review only

**Figure legends**

**Figure 1** A conceptual flow diagram summarising main findings and potential interactions between key themes

Green arrows and boxes represent factors with potential to *positively* impact quality of life; Red arrows and boxes represent factors with potential to *negatively* impact quality of life; A. Reflecting positively upon past experience and psychological attributes may positively impact quality of life following retirement from professional cricket; B. Reflecting positively upon past experience and psychological attributes may reduce the potential for pain, osteoarthritis and physical limitations to negatively impact quality of life after retirement from professional cricket; Together, A. and B. provide one potential explanation for high quality of life despite a high prevelance of pain, osteoarthirits and physical limitations in this sample of retired professional cricketers; Question marks denote uncertainty surrounding the nature of the relationship between cricket-related experiences and psychological attributes common in successful cricketers, since individuals may possess these attributes prior to cricket participation

For peer review only

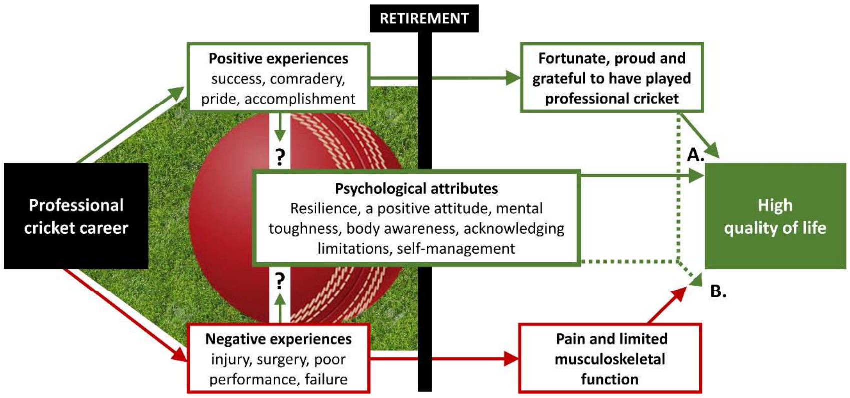


Figure 1 A conceptual flow diagram summarising main findings and potential interactions between key themes‼ ┼ Green arrows and boxes represent factors with potential to positively impact quality of life;‼ ┼ Red arrows and boxes represent factors with potential to negatively impact quality of life;‼ ┼ A. Reflecting positively upon past experience and psychological attributes may positively impact quality of life following retirement from professional cricket;‼ ┼ B. Reflecting positively upon past experience and psychological attributes may reduce the potential for pain, osteoarthritis and physical limitations to negatively impact quality of life after retirement from professional cricket;‼ ┼ Together, A. and B. provide one potential explanation for high quality of life despite a high prevelance of pain, osteoarthirits and physical limitations in this sample of retired professional cricketers;‼ ┼ Question marks denote uncertainty surrounding the nature of the relationship between cricket-related experiences and psychological attributes common in successful cricketers, since individuals may possess these attributes prior to cricket participation‼ ┼

677x381mm (300 x 300 DPI)

For peer review only - http://bmjopen.bmj.com/site/about/guidelines.xhtml

**Supplementary Appendix 1** Semi-structured interview guide

| |
|---|
| 1. Can you describe any physical activity, exercise or sport that you currently take part in? |
| 2. Has that remained fairly constant since you retired from cricket or has it changed over the years? |
| 3. Have you played cricket again since retiring? Why/why not? |
| 4. What was your motivation for playing cricket? |
| 5. Are you as physically active as you would like to be? If no, why not? How does this make you feel? |
| 6. What is your motivation for taking part in physical activity/exercise/sport? |
| 7. How important is being physically active to you? (If important, why is it important? / If not important, has it always been this way?) |
| 8. Does the type of physical activity that you do matter to you, or would you be satisfied taking part in any form of physical activity?<br>(ask about specific forms of exercise that they find *dissatisfying* and why) |
| 9. What physical activity goals are you currently trying to achieve, if any? |
| 10. What are the barriers or challenges, if any, that impact on your ability to be physically active? |
| 11. Do you think that retired cricketers face the same challenges with being physically active as the general population, or are they unique or different in some way? |
| 12. Some retired cricketers become physically inactive, what advice would you give to help them maintain a physically active lifestyle after retiring from cricket? |
| 13. If you wanted to increase your physical activity levels, what do you think would help you to do so? |
| 14. Can you describe any positive or negative impacts that your previous participation in cricket has had upon your current physical activity patterns? |
| 15. If you hadn't played professional cricket, do you think that you would be more or less active, than you currently are? |
| 16. Does your current ability to participate in physical activity impact upon your quality of life? If yes, in what ways? If no, why not? |
| 17. Overall how satisfied are you with your current quality of life?<br>Do you think that this is related to your past career in cricket? |
| 18. Is there anything more you would like to add about your experiences with physical activity after retiring from professional cricket? |

BMJ Open

# Common attributes in retired professional cricketers that may enhance or hinder quality of life after retirement: A qualitative study

| | |
|---|---|
| Journal: | *BMJ Open* |
| Manuscript ID | bmjopen-2017-016541.R2 |
| Article Type: | Research |
| Date Submitted by the Author: | 08-May-2017 |
| Complete List of Authors: | Filbay, Stephanie; University of Oxford, Arthritis Research UK Centre for Sport, Exercise & Osteoarthritis, Nuffield Department of Orthopaedics, Rheumatology & Musculoskeletal Sciences<br>Bishop, Felicity; University of Southampton, Psychology<br>Peirce, Nicholas; Loughborough University, National Centre for Sport and Exercise Medicine & National Cricket Performance Centre<br>Jones, Mary; University of Oxford, Arthritis Research UK Centre for Sport, Exercise & Osteoarthritis, Nuffield Department of Orthopaedics, Rheumatology & Musculoskeletal Sciences<br>Arden, Nigel; University of Oxford, Arthritis Research UK Centre for Sport, Exercise & Osteoarthritis, Nuffield Department of Orthopaedics, Rheumatology & Musculoskeletal Sciences |
| <b>Primary Subject Heading</b>: | Sports and exercise medicine |
| Secondary Subject Heading: | Qualitative research |
| Keywords: | SPORTS MEDICINE, Osteoarthritis, PAIN MANAGEMENT, QUALITATIVE RESEARCH, Resilience |
| | |

SCHOLARONE™
Manuscripts

# Common attributes in retired professional cricketers that may enhance or hinder quality of life after retirement: A qualitative study

Filbay S.R, Bishop F.L, Peirce N, Jones M.E, Arden N.K.

Filbay S.R.

Arthritis Research UK Centre for Sport, Exercise & Osteoarthritis, Nuffield Department of Orthopaedics, Rheumatology & Musculoskeletal Sciences, University of Oxford
Botnar Research Centre, Windmill Rd, Oxford UK OX3 7LD
t: 07 414 064 683 | e: stephanie.filbay@uq.net.au | twitter: @stephfilbay

Bishop F.L.

Psychology department, University of Southampton, Southampton, UK. SO17 1BJ
t: +44 (0)23 8059 9020 | e: F.L.Bishop@southampton.ac.uk

Peirce N.

National Centre for Sport and Exercise Medicine & National Cricket Performance Centre, Loughborough University, Leicester, LE11 3TU UK
t: + 44 (0) 1509 228663 | e: nick.peirce@ecb.co.uk

Jones M.E.

Nuffield Department of Orthopaedics, Rheumatology & Musculoskeletal Sciences, University of Oxford
Botnar Research Centre, Windmill Rd, Oxford UK OX3 7LD
t: 01865 223436 | e: mary.jones@oriel.ox.ac.uk

Arden N.K.

Arthritis Research UK Centre for Sport, Exercise & Osteoarthritis, Nuffield Department of Orthopaedics, Rheumatology & Musculoskeletal Sciences, University of Oxford
Botnar Research Centre, Windmill Rd, Oxford UK OX3 7LD
t: +44 (0)1865 737859 | e: nigel.arden@ndorms.ox.ac.uk



For peer review only - http://bmjopen.bmj.com/site/about/guidelines.xhtml

**Word count** 3984 (4000 Word Limit)

# ABSTRACT


## Objectives

Retired professional cricketers shared unique experiences and may possess specific psychological attributes with potential to influence quality of life (QOL). Additionally, pain and osteoarthritis can be common in retired athletes which may negatively impact QOL. However, QOL in retired athletes is poorly understood.

This study explores the following questions from the personal perspective of retired-cricketers: How do retired cricketers perceive and experience musculoskeletal pain and function in daily life? Are there any psychological attributes that might enhance or hinder retired cricketers' QOL?

## Design

A qualitative study using semi-structured interviews, which were subject to inductive, thematic analysis. A data-driven, iterative approach to data coding was employed.

## Setting

All participants had lived and played professional cricket in the United Kingdom and were living in the UK or abroad at the time of interview.

## Participants




For peer review only - http://bmjopen.bmj.com/site/about/guidelines.xhtml

18 male participants, aged a mean 57±11 (range 34 to 77) years had played professional cricket for a mean 12±7 seasons and had been retired from professional cricket on average 23±9 years.

## Results

Fifteen participants reported pain or joint difficulties and all but one was satisfied with their QOL. Most retired cricketers reflected upon experiences during their cricket career that may be associated with the psychological attributes that these individuals shared, including resilience and a positive attitude. Additional attributes included a high sense of body awareness, an ability to self-manage pain and adapt lifestyle choices to accommodate physical limitations. Participants felt fortunate and proud to have played professional cricket, which may have further contributed to the high QOL in this group of retired cricketers.

## Conclusions

Most retired cricketers in this study were living with pain or joint difficulties. Despite this, all but one was satisfied or very satisfied with their QOL. This may be partly explained by the positive psychological attributes that these retired cricketers shared.

For peer review only

## Strengths and limitations of this study

- A strength of this study was the strong themes and consistent patterns that emerged through the interviews and the deep reflection and open, personal experiences that retired-cricketers shared that enriched the findings of this study.
- Since participants had been retired from professional cricket for an average of 23 years, we gained unique insight into longer-term quality of life after professional sport which had not been previously explored.
- It is not known whether cricket participation contributed to the development of positive psychological attributes or whether these largely pre-existed prior to cricket participation or developed after retirement.
- Limitations include generalisability and the possibility of selection bias, whereby retired cricketers with specific characteristics may be more likely to desire participation in a qualitative interview study.

## Funding

Dr Filbay was awarded a research fellowship from the Arthritis Research UK Centre for Sport, Exercise and Osteoarthritis to support this research. Dr Arden and Mary Jones have received an unrestricted research grant from the England and Wales Cricket Board. The parent cross-sectional study from which participants were recruited received funding from the Arthritis Research UK Centre for Sport, Exercise and Osteoarthritis, as well as the England and Wales Cricket Board.

## Competing interests

For peer review only

Dr Arden and Mary Jones have received an unrestricted research grant from the England and Wales Cricket Board. Dr Peirce is employed as the Chief Medical Officer of the England and Wales Cricket Board. Dr Filbay and Dr Bishop have nothing to disclose.

For peer review only



For peer review only - http://bmjopen.bmj.com/site/about/guidelines.xhtml

## Introduction

Research surrounding the long-term outcomes of a professional sporting career has largely focused on negative impacts rather than investigating the potential for positive psychological impacts that persist beyond retirement.[1] On retirement, athletes commonly experience negative emotions and reduced life satisfaction while transitioning and adapting to an alternative lifestyle,[2] but very little is known about life after this transition. After retirement, athletes are at increased risk of developing osteoarthritis, predominately due to a history of sport-related injury, and this can result in pain and activity limitations.[3-7] However, retired athletes may possess psychological attributes that could enhance quality of life and facilitate effective coping with pain and musculoskeletal impairment. Additionally, retired athletes may feel proud and accomplished to have played professional sport which could have positive impacts on QOL. On the other hand, functional limitations in individuals with high athletic identity could negatively impact QOL if their physical activity desires are not satisfied. These complex possible influences on the QOL of retired athletes are poorly understood.

Professional cricket is all-encompassing, the duration of play exceeds most other sports with formats including entire day matches and test matches lasting up to five consecutive days. In cricket, different playing positions have distinct psychological demands. A batsman facing a ball is under great pressure to perform, one slip of concentration or an incorrect decision can see him out of the game with no further opportunity to contribute positively to the team. A fielder in a slip position maintains prolonged intense focus in preparation for a potential catch with an outcome of success or failure determined by a momentary reaction. The demands of cricket contrast most other team sports where players have greater opportunity to correct



For peer review only - http://bmjopen.bmj.com/site/about/guidelines.xhtml

performance and ongoing involvement within a game. Thus, professional cricketers face unique competitive demands that require advanced psychological and behavioural control to manage stress and perform under pressure.[8 9] The ability to deal with such stressors will in part, determine the success of a professional cricketer. Psychological attributes associated with a successful cricket career include resilience, mental toughness, self-belief, optimism, coping with adversity and confidence.[10-12] Although these psychological attributes have been observed in professional cricketers, it is not known whether they persist after retirement. The psychological attributes that retired cricketers possess have potential to benefit other aspects of life and reduce the impact of musculoskeletal pain following retirement from cricket.

We undertook a qualitative study to provide an in-depth analysis of life after retirement from professional cricket, from the personal perspective of retired-cricketers. The a priori aim of this qualitative study was to explore physical activity after retirement from professional cricket. However, the study also captured participants' broader perspectives regarding QOL and prominent themes linking QOL to professional cricket emerged during the initial inductive analysis. This paper focuses on these emergent themes, which were explored while considering two sensitising questions: How do retired cricketers perceive and experience musculoskeletal pain and function in daily life? Are there any psychological attributes that might enhance or hinder retired cricketers' QOL? A complementary manuscript will address physical activity after retirement from professional cricket.

## Methods

The study methodology and results are reported in accordance with the consolidated criteria for reporting qualitative research (COREQ) guidelines.[13]



For peer review only - http://bmjopen.bmj.com/site/about/guidelines.xhtml

***Ethical considerations***

This study has been approved by Medical Sciences Inter-divisional Research Ethics Committee (IDREC), University of Oxford (reference number R45197/RE001).

***Participants and recruitment***

Participants were purposively sampled from a larger cohort study of 187 retired professional English cricketers who recently completed a cross-sectional questionnaire collecting information on demographics, playing history, current pain, medical history (including doctor diagnosed osteoarthritis, arthroscopic surgery and joint replacement surgery) and injury history (Jones et al. *under review*). In order to facilitate a balanced exploration of physical activity behaviours, barriers, and facilitators, we purposively sampled individuals based on their responses to two questions from the cross-sectional study assessing perceived impact of previous cricket participation upon current physical activity levels: i) Strongly agree or agree that participation in cricket has resulted in an *increase* in current physical activity level (n=46, 42%), or ii) Strongly agree or agree that participation in cricket has resulted in a *decrease* in current physical activity levels (n= 27, 25%). We aimed to include an equal number of participants from each of these groups.

Only individuals from the larger cross-sectional study who indicated a willingness to participate in future sport-related research were invited (by email) into the current study. The invitation email included an information sheet describing the study purpose, procedure (including data storage and assurance of confidentiality), dissemination plan and the interviewer's credentials. Among those expressing an interest in this study, participants were then purposively sampled to ensure men of a range of ages were interviewed. Interviews were



For peer review only - http://bmjopen.bmj.com/site/about/guidelines.xhtml

conducted until the point of data saturation, defined a priori as the point at which no new themes were identified from four consecutive interviews (two from participants with reduced physical activity and two from participants with increased physical activity). After which, additional interviews were performed as deemed appropriate to explore ideas and themes in greater detail after following the semi-structured interview guide. These final interviews were also used to affirm data saturation. If new themes emerged from these final interviews, additional interviews would be conducted until data saturation is satisfied.

A total of 42 email invitations were sent to eligible participants, 19 emails received no response, 2 individuals declined to participate, 2 individuals were unavailable due to overseas travel and 1 individual indicated a willingness to participate but did not respond to further correspondence. Data saturation was achieved by the 14th interview. An additional four interviews were carried out after data saturation and no new themes emerged. Data from all 18 interviews were used for analysis.

***Interviews***

Audio recorded semi-structured telephone interviews were performed by S.R.F (a female postdoctoral researcher and physiotherapist with experience in qualitative research). The interviewer was unknown to participants prior to the interview. Informed verbal consent was obtained from each participant prior to taking part. Interviews were transcribed verbatim by a research assistant. All transcripts were de-identified during transcription, and an alias was assigned to each participant. The 18 interviews were on average 26 minutes in length (ranging from 18 to 37 minutes).



For peer review only - http://bmjopen.bmj.com/site/about/guidelines.xhtml

A semi-structured interview guide was used to assure that key areas of interest were covered while enabling the researcher to adapt the interview guide to elicit relevant and rich information from respondents by probing and prompting for additional details.[14] The interview guide was pilot tested with three individuals with cricket experience prior to gaining ethics approval. The interview guide contained open-ended questions to provide participants with the greatest opportunity to consider personal perspectives and experiences (Appendix 1). The interview guide was iteratively adapted throughout the interviews to incorporate any additional issues of importance to respondents (for example, by adding a question to explore their relationship with cricket post-retirement). Interviewees were given the opportunity to contribute any additional information at the end of the interview.

***Analysis procedure***

An inductive thematic approach was used[15 16] facilitated by NVivo version 11 software.[17] Following each interview, the interviewer summarised and reflected upon initial ideas in a study journal. Transcripts were read and re-read with accompanying audio to identify all information relevant to the research aims.[18] An iterative approach to data coding was employed. All data that might be relevant to the aims of the study were coded into multiple categories to be refined and analysed for themes in subsequent stages of analysis.[15] During the earlier stages of analysis, no attention was given to the prevalence of themes and coding was performed diversely and inclusively, withholding further interpretation. In line with an inductive thematic approach, coding was data-driven, performed without reference to a pre-existing coding structure and with little engagement with literature to avoid sensitization to specific themes.[15 18]


For peer review only - http://bmjopen.bmj.com/site/about/guidelines.xhtml

During the later stages of analysis, the data was further analysed for repeated patterns, codes were sorted into a hierarchical structure representing themes and subthemes, overlapping themes were merged, and those outside the scope of the current study were filed separately. These themes and sub-themes were repeatedly reviewed and refined to confirm external heterogeneity and internal homogeneity within themes and to ensure an accurate representation of the entire dataset.[15 19] During this stage, all transcripts were re-read and the study journal was revisited to check that themes accurately reflected the key issues discussed by participants. A selection of six transcripts representing participants with diverse physical activity patterns were analysed by a second investigator (F.L.B) blinded to the coding structure developed by the first author (S.R.F). A meeting was then held to discuss key themes, and a high level of agreement was achieved between investigators. Key themes and sub-themes will be described with reference to participant quotes.[15 19]

Following qualitative analysis, responses to relevant questions collected as part of the cross-sectional study were used to describe the study sample (including osteoarthritis status, age, previous surgery and length of professional cricket career). Questionnaire data was cross-checked for accuracy with participants' narratives and if disparities were present (e.g. a participant reported no current joint pain on questionnaire but described experiencing current joint pain during the interview), participant narratives were considered more reliable.

## Results

### *Participant characteristics*



For peer review only - http://bmjopen.bmj.com/site/about/guidelines.xhtml

The 18 participants who took part in the interviews were all male, aged a mean 57±11 (range 34 to 77) years, played professional cricket in the United Kingdom for a mean 12±7 (range 2 to 25) seasons and had been retired from professional cricket for an average 23±9 (range 7 to 38) years. The majority were overweight by World Health Organisation (WHO) criteria[20] (n=12, 67%) and three participants (17%) were classified as obese. Current joint pain or difficulties were common (n=15, 83%), 10 people (56%) had been told by a doctor that they had osteoarthritis, 12 participants (67%) had received at least one joint surgery and 5 participants (28%) had received one or more total joint replacement. More than two thirds (n=14, 78%) reported having ever had an injury that resulted in more than one month of reduced participation in exercise, training or sport. Full participant characteristics are reported in Table 1.

[Insert Table 1]

***Pain and musculoskeletal function***

Fifteen retired cricketers described experiencing current pain and limitations with musculoskeletal function. For most individuals, this prevented them for taking part in specific activities, like running and recreational sports. Although this was described as frustrating by some, participants had made lifestyle modifications to accommodate these impairments and very few described a negative impact upon QOL.

> *Jim: 'I've had 13 operations over the years because of cricket injuries and resulting stuff from that and I'm just restricted in what I can do really. ..it doesn't make me feel, nothing really, as long as I can do the little bits I can I'm quite happy.'*

> *Sam: 'So there are little bits that frustrate me but I mean, I'm nearly 60 so you know, your body does get worn out if you do something as extreme as play a professional*



For peer review only - http://bmjopen.bmj.com/site/about/guidelines.xhtml

*sport for 17 years, so yeah they're just things that exist, but no, I'm not that bothered.'*

***The ups and downs of professional cricket***

Retired-cricketers described experiencing mental hardship and psychological challenges during their cricket career. These included coming to terms with experiencing more failure than success and difficulty coping with pressure to perform. Almost all participants believed these experiences benefited other areas of life after retirement. Specifically, they described how attributes required to succeed on the cricket field, including mental toughness, confidence, and resilience, equipped them with tools to overcome post-retirement life challenges, including managing chronic pain and establishing a successful career outside of cricket.

> *Lee 'It was just a constant battle, it was soul destroying at times but out of all that you know; hard work, disappointment, you know, being beaten down by people at times… I think it's given me the mental capacity just to, just get your head down and battle on. I think that's the main thing, you can have bad times in whatever you're doing, and you're going to feel really low, but I think just that mental capacity that you've learnt from cricket, from being a professional sportsmen, to just get back up on your feet and fight another day sort of thing is being part of where I've got to now, you know thanks to that basically.'*

Only one individual felt that the mental aspects of professional cricket had a negative impact upon life after retirement. Dan attributed difficulty coping with adversity and reduced confidence to having played professional cricket.

> *Dan: 'I wouldn't say I carry mental scars, but I think it's affected my personality.. ..probably I am less confident having been a professional sportsman than I would have been otherwise, because I think I have been beaten up quite a few times over the years, you know mentally, and it's taken its toll a little bit.'*

***Psychological attributes that may enhance quality of life***



For peer review only - http://bmjopen.bmj.com/site/about/guidelines.xhtml

*Resilience and a positive attitude*

Attributes that appeared to enhance QOL in this sample of retired cricketers, included resilience and a positive attitude. Almost all retired-cricketers in this study demonstrated characteristics and described experiences suggesting high levels of resilience and a positive attitude regarding pain and musculoskeletal impairment.

> *Dom: 'I would classify myself to be very, very lucky, probably very, very lucky to come out the other side, with just, at the moment, hip injuries that have been cured by hip operations… it's all been extremely positive, from a life style I've reached, yeah.'*

> *Ron: 'Keep a strong mind and you will have aliments depending on how much sport you have played and you just have to get on with it.''*

*Body awareness, acknowledging limitations and self-management*

There were other common attributes amongst the retired cricketers that appeared to have a positive impact upon QOL, these included a heightened sense of body awareness and confidence in self-managing physical ailments. Most participants had made positive adaptations and were mindful of their body's capabilities. Participants adhered to self-imposed activity limits and modified their activity choices on a day to day basis to avoid exacerbation of pain or an acute deterioration in physical function.

> *Fin: '.. you also have a greater and wiser knowledge of what you should, could, and can and can't do. So from a positive point of view you know what you can and can't do and you know what's good for you and your body.'*

> *Gus: 'I like to think that I know what my, what my boundaries are and I can just push them a little bit or to the limit that I think that, that my age will allow me to do... I definitely think that playing cricket made me certainly more aware and being involved in cricket for the amount of time that I had, I certainly have, have more knowledge of what I can and can't do.'*

*Fortunate, proud and grateful to have played professional cricket*



For peer review only - http://bmjopen.bmj.com/site/about/guidelines.xhtml

Reflecting upon their past career in cricket, all participants felt fortunate, proud or grateful to have had the opportunity to play professional cricket. All but one individual believed that overall, the positive effects of cricket outweighed the negative, and expressed no regret over playing professional cricket.

> *Fin: 'No regrets what so ever. It was a great life. I had a great time. There were things that, when you come out of that sporting environment into the real world, if you like, and you realise that then its only things that other people dream of doing, is what you've done. So absolutely no regrets what so ever.'*

> *Sam: 'You just play the hand your dealt and I never think there's any point having any regrets or bearing any grudges, it is what it is and you know I've had a great life out of it. …I've made plenty of rubbish decisions, but I wouldn't change that fact, because all that failure and all that success has made me who I am and I'm quite happy being me now.'*

***Quality of life***

Irrespective of pain and joint difficulties, most retired-cricketers were very satisfied (n=11, 61%) or satisfied (n=6, 33%) with their QOL. No participants expressed that their QOL was impaired due to pain or physical issues. The only individual dissatisfied with his QOL attributed this entirely to current family difficulties.

> Interviewer: Overall how satisfied are you with your current quality of life?

> *Wes: '110%, cannot believe my luck. ..I am spoilt rotten, but I mean there's no point in being in a brilliant position, which I am, if you don't appreciate it, I mean I can see lots of people who don't know how lucky they are, well, I am glad to say I do.*

> *Sam: 'I am absolutely delighted with it, I love it. I'm in a really good place mentally… things couldn't be better.'*

> *Leo: 'Oh brilliant, I'm just very, very lucky, absolutely brilliant.'*

> *Ken: 'I am very happy with it actually, and have been for a number of years.'*

> *Joe: 'Good, yeah I'm very happy.'*

> *Guy: 'Oh on a scale, I would say 8/9 out of 10; I think I am very satisfied.'*



For peer review only - http://bmjopen.bmj.com/site/about/guidelines.xhtml

*Gus: 'Well I am very happy, very happy and pleased with my quality of life.'*

*Dom: 'Very happy. ..very happy with where I am physically and mentally in what I do and what I want to do, yes.'*

*Ned: 'Oh generally, you know, very happy really, I think you know keeping things in perspective of, you know of how I am, my age, you know, what I've got, you know I can't complain really. I am really pretty pleased with the way things are.'*

***Summary of key themes***

Figure 1 provides a conceptual flow diagram summarising the main findings and providing an overview of how the key themes may interact to make a positive contribution to QOL after retirement.

[Insert Figure 1 here]

## Discussion

Current pain and physical limitations were common in this group of retired professional cricketers. Despite this, almost all retired cricketers were satisfied or very satisfied with their QOL. One potential explanation for this counterintuitive finding is the common psychological attributes that these individuals may possess, including resilience, a positive attitude, heightened body awareness, acknowledgement of physical limitations, and an ability to modify activity choices to effectively cope with fluctuations in pain and musculoskeletal function. Reflecting upon their past cricket career, participants felt very grateful and fortunate to have played professional cricket, and all but one felt the positive impacts of a career in cricket outweighed any negatives. This feeling of pride and accomplishment has further



For peer review only - http://bmjopen.bmj.com/site/about/guidelines.xhtml

potential to positively influence QOL in this group of retired cricketers, irrespective of pain and physical impairment.

***Quality of life and flourishing***

We found that all but one retired-cricketer was satisfied with his QOL. Flourishing is a concept that represents an optimal QOL where an individual excels emotionally, psychologically and socially. In 2013, 20% of a representative sample of the UK general population were found to be 'flourishing' (possessing 9 of 10 signs of positive wellbeing including resilience, optimism, competence, self-esteem and meaning).[21] Retired cricketers who took part in the interviews exhibited many attributes consistent with the definition of flourishing. It is possible that a greater number of retired-cricketers 'flourish' in later life compared with the general population despite a higher prevalence of pain and osteoarthritis, although further research is necessary to explore this possibility.

Retired professional footballers with osteoarthritis reported worse health-related QOL compared with retired footballers without osteoarthritis.[22] However, most conventional QOL outcome measures (including the EQ-5D used in the study of retired footballers[22]) are influenced by pain and physical impairment, where the presence of pain and physical disability will result in a reduced QOL score, irrespective of the impact of pain or disability upon the individual.[23] Evaluation of the psychometric properties of QOL measures in retired sporting populations is warranted. The Flourishing Scale[24] or a QOL measure not driven by pain and function (such as the WHO-QOL[25]) may be preferable for use in retired sporting populations.



For peer review only - http://bmjopen.bmj.com/site/about/guidelines.xhtml

***Resilience and associated positive psychological attributes***

Resilience refers to responding to adversity with positive adaptation, and in doing so, restoring an internal sense of emotional and psychological balance.[26 27] Retired-cricketers in the present study shared resilient attributes, this was highlighted by their ability to maintain a positive attitude and adapt activity choices to effectively cope with fluctuations in pain and physical function. Prior research has found that athletes possess greater resilience and optimism than non-athletes[28] and successful cricketers are likely to be more resilient than less successful cricketers.[10 12] A high level of resilience is associated with increased QOL in a wide variety of disease-specific and chronic pain populations.[29-36] Resilient individuals also demonstrate more adaptive coping styles and better musculoskeletal pain adjustment.[37 38] Considering sporting injury is a risk factor for osteoarthritis, interventions to build resilience within a sporting setting[39] may have benefits that extend beyond success on the sporting field, with potential to enhance QOL in later life.

Despite these benefits, who is likely to become resilient and why is poorly understood.[40] The relationship between resilience and professional cricket is likely multifaceted. Exposure to the demands of cricket from a young age, including repetitive failure, disappointment, stress and adversity may facilitate the development of resilience. On the other hand, resilience may be influenced by a range of non-cricket related factors, and resilient individuals may be most likely to reach professional status resulting in a high level of resilience in professional cricketers. It is also possible that adversities faced during transition from sport and after retirement contributed to the high level of resilience in this group of retired cricketers.

***Cricket reflection***



For peer review only - http://bmjopen.bmj.com/site/about/guidelines.xhtml

Accomplishment refers to success, achievement or mastery at the highest possible level[41] and has been recognised as an important component of wellbeing.[42] Considering all retired-cricketers in this study were fortunate, proud or grateful to have played professional cricket it is possible that this accomplishment further contributed to the high QOL described by these individuals. A retired athlete's sense of accomplishment and fulfilment through past participation in sport is too often overlooked in research, despite potential to positively influence QOL.

***Study strengths and potential limitations***

It is not known whether cricket participation contributed to the development of positive psychological attributes or whether these largely pre-existed prior to cricket participation or developed after retirement. Prospective longitudinal studies would be required to answer this question. Although participants were asked about their degree of satisfaction with their overall QOL, we did not explore personal beliefs about the definition of QOL. Consequently the question pertaining to QOL could have meant different things to different participants. Due to purposive recruitment, individuals reporting uncertainty regarding the impact of cricket upon their physical activity level were not invited into the study, reducing the generalisability of results. It is also possible that the study was subjected to selection bias, whereby retired cricketers with specific characteristics were more likely to desire participation in a qualitative interview study. Repeat interviews were not performed and transcripts were not returned to participants for correction or comment, these procedures could have provided additional information and insights beyond those gained through the initial interviews. A strength of this study was the strong themes and consistent patterns that emerged through the interviews resulting in saturation of data at 14 interviews, confirmed by



For peer review only - http://bmjopen.bmj.com/site/about/guidelines.xhtml

the final four interviews that enabled further complimentary exploration into ideas and important themes. The interviewer was a physiotherapist with experience in building rapport with individuals and capturing issues of importance to an individual. Rapport was further strengthened by the interviewer's familiarity with cricket as a physiotherapist and cricket participant. These factors facilitated deep reflection and open, personal insights from retired-cricketers that enriched the findings of this study.

## Conclusion

Irrespective of chronic joint pain and physical limitations, all but one retired-cricketer in this study was satisfied or very satisfied with their QOL. Retired-cricketers shared common psychological attributes including resilience and a positive attitude that may partly explain the small impact pain and physical limitations appeared to have upon the QOL of these retired cricketers. Additionally, most retired cricketers had a high sense of body awareness and executed self-control to adapt activity choices and accept physical limitations. Participants felt fortunate and grateful to have played professional cricket and expressed that the benefits of cricket participation outweigh any negative impacts. This study highlights fruitful areas for future research including i) flourishing in retired athletes despite pain and functional limitations, ii) the potential for positive psychological attributes to develop through sport participation and impact QOL after retirement, and iii) the effect of past accomplishment in sport on QOL across the lifespan. A better understanding of the relationship between sport participation and QOL could assist in promoting physical activity and facilitate strategies to enhance the positive impact of sport upon QOL.

## Acknowledgments



For peer review only - http://bmjopen.bmj.com/site/about/guidelines.xhtml

388 We would like to thank Angus Porter and the Professional Cricketers' Association (PCA) for
389 assisting with recruitment and questionnaire development for the larger cross-sectional study
390 from which study participants were purposively recruited.

391

## 392 Author contributions

393 SRF, FLB, NP, NKA conceived and designed this qualitative study. SRF, MEJ recruited
394 participants and extracted data form the cross-sectional cohort. SRF performed all interviews.
395 SRF, FLB participated in the analysis. SRF drafted the first version of the manuscript. All
396 authors contributed in revising the manuscript and gave their final approval of the submitted
397 version.

398

## 399 Data sharing

400 To view interview transcripts or additional participant quotes, please contact the
401 corresponding author.



For peer review only - http://bmjopen.bmj.com/site/about/guidelines.xhtml

## References

1. Knights S, Sherry E, Ruddock-Hudson M. Investigating Elite End-of-Athletic-Career Transition: A Systematic Review. *Journal of Applied Sport Psychology* 2016:1-18. doi: 10.1080/10413200.2015.1128992
2. Park S, Lavallee D, Tod D. Athletes' career transition out of sport: a systematic review. *International Review of Sport and Exercise Psychology* 2013;6(1):22-53. doi: 10.1080/1750984X.2012.687053
3. Spector T, Harris P, Hart DJ, et al. Risk of osteoarthritis associated with long-term weight-bearing sports. *Arthritis and rheumatism* 1996;39(6):988-95.
4. Thelin N, Holmberg S, Thelin A. Knee injuries account for the sports-related increased risk of knee osteoarthritis. *Scandinavian journal of medicine & science in sports* 2006;16(5):329-33.
5. Gelber AC, Hochberg MC, Mead LA, et al. Joint injury in young adults and risk for subsequent knee and hip osteoarthritis. *Annals of internal medicine* 2000;133(5):321-28.
6. Gouttebarge V, Inklaar H, Backx F, et al. Prevalence of osteoarthritis in former elite athletes: a systematic overview of the recent literature. *Rheumatology international* 2015;35(3):405-18. doi: 10.1007/s00296-014-3093-0 [published Online First: 2014/07/20]
7. Arliani GG, Astur DC, Yamada RKF, et al. Early osteoarthritis and reduced quality of life after retirement in former professional soccer players. *Clinics* 2014;69(9):589-94. doi: 10.6061/clinics/2014(09)03
8. Neil R, Bowles HCR, Fleming S, et al. The experience of competition stress and emotions in cricket. *Sport Psychologist* 2016;30(1):76-88. doi: 10.1123/tsp.2014-0077
9. Thelwell RC, Weston NJ, Greenlees IA. Batting on a sticky wicket: Identifying sources of stress and associated coping strategies for professional cricket batsmen. *Psychology of Sport and Exercise* 2007;8(2):219-32.
10. Bull SJ, Shambrook CJ, James W, et al. Towards an understanding of mental toughness in elite English cricketers. *Journal of Applied Sport Psychology* 2005;17(3):209-27. doi: 10.1080/10413200591010085
11. Weissensteiner J, Abernethy B, Farrow D. Towards the development of a conceptual model of expertise in cricket batting: A grounded theory approach. *Journal of Applied Sport Psychology* 2009;21(3):276-92. doi: 10.1080/10413200903018675
12. Weissensteiner JR, Abernethy B, Farrow D, et al. Distinguishing psychological characteristics of expert cricket batsmen. *Journal of Science and Medicine in Sport* 2012;15(1):74-79.
13. Tong A, Sainsbury P, Craig J. Consolidated criteria for reporting qualitative research (COREQ): a 32-item checklist for interviews and focus groups. *International journal for quality in health care : journal of the International Society for Quality in Health Care* 2007;19(6):349-57. doi: 10.1093/intqhc/mzm042 [published Online First: 2007/09/18]
14. Jones I, Brown L, Holloway I. Qualitative Research in Sport and Physical Activity: SAGE Publications 2012.
15. Braun V, Clarke V. Using thematic analysis in psychology. *Qualitative Research in Psychology* 2006;3(2):77-101. doi: 10.1191/1478088706qp063oa
16. Thomas DR. A General Inductive Approach for Analyzing Qualitative Evaluation Data. *American Journal of Evaluation* 2006;27(2):237-46. doi: 10.1177/1098214005283748
17. Bazeley P, Jackson K. Qualitative data analysis with NVivo. Los Angeles: SAGE 2013.
18. Tuckett AG. Applying thematic analysis theory to practice: A researcher’s experience. *Contemporary Nurse* 2005;19(1-2):75-87. doi: 10.5172/conu.19.1-2.75
19. Patton MQ. Qualitative evaluation and research methods1990.
20. Organisation WH. Obesity: Preventing and managing the global epidemic. Report of a WHO Consultation on Obesity, Geneva,. *WHO/NUT /NCD/98 1, Geneva, 1998* 3-5 June 1997



For peer review only - http://bmjopen.bmj.com/site/about/guidelines.xhtml

21. Huppert FA, So TTC. Flourishing Across Europe: Application of a New Conceptual Framework for Defining Well-Being. *Social Indicators Research* 2013;110(3):837-61. doi: 10.1007/s11205-011-9966-7
22. Turner AP, Barlow JH, Heathcote-Elliot C. Long term health impact of playing professional football in the United Kingdom. *British Journal of Sports Medicine* 2000;34(5):332-37. doi: 10.1136/bjsm.34.5.332
23. Filbay SR. Longer-term quality of life following anterior cruciate ligament injury and reconstruction. *PhD Thesis, School of Health and Rehabilitation Sciences, The University of Queensland* 2016;1st July 2016 doi: 10.14264/uql.2016.384
24. Diener E, Wirtz D, Tov W, et al. New Well-being Measures: Short Scales to Assess Flourishing and Positive and Negative Feelings. *Social Indicators Research* 2010;97(2):143-56. doi: 10.1007/s11205-009-9493-y
25. Group TW. Development of the World Health Organization WHOQOL-BREF Quality of Life Assessment. *Psychological medicine* 1998;28(3):551-58. doi: 10.1017/S0033291798006667
26. Galli N, Gonzalez SP. Psychological resilience in sport: A review of the literature and implications for research and practice. *International Journal of Sport and Exercise Psychology* 2015;13(3):243-57. doi: 10.1080/1612197X.2014.946947
27. Schinke RJ, Battochio RC, Dube TV, et al. Adaptation Processes Affecting Performance in Elite Sport. *Journal of Clinical Sport Psychology* 2012;6(2):180-95. doi: doi:10.1123/jcsp.6.2.180
28. Guillén F, Laborde S. Higher-order structure of mental toughness and the analysis of latent mean differences between athletes from 34 disciplines and non-athletes. *Personality and Individual Differences* 2014;60:30-35. doi: 10.1016/j.paid.2013.11.019
29. Li MY, Yang YL, Liu L, et al. Effects of social support, hope and resilience on quality of life among Chinese bladder cancer patients: A cross-sectional study. *Health and Quality of Life Outcomes* 2016;14(1) doi: 10.1186/s12955-016-0481-z
30. Ring A, Jacoby A, Baker GA, et al. Does the concept of resilience contribute to understanding good quality of life in the context of epilepsy? *Epilepsy and Behavior* 2016;56:153-64. doi: 10.1016/j.yebeh.2016.01.002
31. Wu WW, Tsai SY, Liang SY, et al. The Mediating Role of Resilience on Quality of Life and Cancer Symptom Distress in Adolescent Patients With Cancer. *Journal of Pediatric Oncology Nursing* 2015;32(5):304-13. doi: 10.1177/1043454214563758
32. Vitali SA. Finding quality of life despite MS: Harnessing resilience. *International MS Journal* 2011;17(3):94-99.
33. Frigiola A, Bull C, Wray J. Exercise capacity, quality of life, and resilience after repair of tetralogy of Fallot: A cross-sectional study of patients operated between 1964 and 2009. *Cardiology in the Young* 2014;24(1):79-86. doi: 10.1017/S1047951112002296
34. Manne SL, Myers-Virtue S, Kashy D, et al. Resilience, Positive Coping, and Quality of Life among Women Newly Diagnosed with Gynecological Cancers. *Cancer Nursing* 2015;38(5):375-82. doi: 10.1097/NCC.0000000000000215
35. Yazdi-Ravandi S, Taslimi Z, Saberi H, et al. The role of resilience and age on quality of life in patients with pain disorders. *Basic and Clinical Neuroscience* 2013;4(1):24-30. doi: 10.1001/archinte.163.20.2433
36. Bergman S, Jacobsson LT, Herrstrom P, et al. Health status as measured by SF-36 reflects changes and predicts outcome in chronic musculoskeletal pain: a 3-year follow up study in the general population. *Pain* 2004;108(1-2):115-23. doi: 10.1016/j.pain.2003.12.013 [published Online First: 2004/04/28]
37. Hildon Z, Montgomery SM, Blane D, et al. Examining Resilience of Quality of Life in the Face of Health-Related and Psychosocial Adversity at Older Ages: What is "Right" About the Way We Age? *The Gerontologist* 2009 doi: 10.1093/geront/gnp067
38. Ruiz-Parraga GT, Lopez-Martinez AE, Gomez-Perez L. Factor structure and psychometric properties of the resilience scale in a spanish chronic musculoskeletal pain sample. *The*



For peer review only - http://bmjopen.bmj.com/site/about/guidelines.xhtml

*journal of pain : official journal of the American Pain Society* 2012;13(11):1090-8. doi: 10.1016/j.jpain.2012.08.005 [published Online First: 2012/10/16]

39. Gonzalez SP, Detling N, Galli NA. Case studies of developing resilience in elite sport: Applying theory to guide interventions. *Journal of Sport Psychology in Action* 2016:1-12. doi: 10.1080/21520704.2016.1236050
40. Elliott AM, Burton CD, Hannaford PC. Resilience does matter: evidence from a 10-year cohort record linkage study. *BMJ Open* 2014;4(1) doi: 10.1136/bmjopen-2013-003917
41. Ericsson KA. Attaining excellence through deliberate practice: Insights from the study of expert performance. *Teaching and learning: The essential readings* 2002:4-37.
42. Forgeard M. J. C. J, E., Kern, M. L., Seligman, M. E. P. Doing the right thing: Measuring wellbeing for public policy. *International Journal of Wellbeing* 2011;1(1):79-106. doi: 10.5502/ijw.v1i1.15

For peer review only



For peer review only - http://bmjopen.bmj.com/site/about/guidelines.xhtml

**Table 1** Participant characteristics

| Alias | Age range[1] | BMI | Joint pain | OA | Past injury | Arthroscopic joint surgery | Joint replacement | Years post retirement[1] | UK professional seasons[1] | Considering the benefits and risks of cricket I would do the same again | I would recommend cricket to significant others |
|---|---|---|---|---|---|---|---|---|---|---|---|
| Cam | 51 to 55 | Overweight | Y | N | Y | N | N | 26 to 30 | 1 to 5 | strongly agree | strongly agree |
| Dan | 56 to 60 | Normal | Y | Y | Y | Y | N | 26 to 30 | 6 to 10 | strongly agree | strongly agree |
| Dom | 61 to 65 | Obese | N | Y | N | Y | Y | 26 to 30 | 16 to 20 | strongly agree | strongly agree |
| Gus | 56 to 60 | Overweight | N | N | N | N | N | 11 to 15 | 1 to 5 | strongly agree | strongly agree |
| Joe | 61 to 65 | Overweight | N | N | Y | N | N | 31 to 35 | 16 to 20 | agree | agree |
| Leo | 76 to 80 | Normal | Y | Y | Y | Y | Y | 36 to 40 | 1 to 5 | strongly agree | strongly agree |
| Ned | 56 to 60 | Overweight | Y | N | N | N | N | 16 to 20 | 16 to 20 | strongly agree | strongly agree |
| Tim | 36 to 40 | Overweight | Y | Y | Y | Y | N | 6 to 10 | NR | strongly agree | strongly agree |
| Wes | 66 to 70 | Overweight | Y | N | Y | N | N | 26 to 30 | 21 to 25 | strongly agree | strongly agree |
| Ben | 56 to 60 | Overweight | Y | Y | Y | Y | Y | 21 to 25 | 11 to 15 | strongly agree | strongly agree |
| Fin | 31 to 35 | Overweight | Y | N | Y | Y | N | 6 to 10 | 6 to 10 | strongly agree | agree |
| Guy | 46 to 50 | Obese | Y | N | Y | Y | N | 21 to 25 | 1 to 5 | strongly agree | strongly agree |
| Jim | 66 to 70 | Overweight | Y | Y | Y | Y | Y | 21 to 25 | 21 to 25 | strongly agree | strongly agree |
| Ken | 56 to 60 | Overweight | Y | Y | Y | N | N | 26 to 30 | 6 to 10 | strongly agree | strongly agree |
| Lee | 46 to 50 | Overweight | Y | N | N | Y | N | 11 to 15 | 6 to 10 | strongly agree | strongly agree |
| Ric | 66 to 70 | Obese | Y | Y | Y | N | Y | 16 to 20 | 1 to 5 | agree | agree |
| Ron | 51 to 55 | Normal | Y | Y | Y | Y | N | 16 to 20 | 16 to 20 | undecided | undecided |
| Sam | 56 to 60 | Overweight | Y | Y | Y | Y | N | 21 to 25 | 16 to 20 | strongly agree | strongly agree |

Participants above the horizontal line strongly agreed or agreed that participation in cricket resulted in an increase in current physical activity level, participants below the horizontal line strongly agreed or agreed that participation in cricket resulted in a decrease in current physical activity levels; [1] Ranges were reported rather than absolute values to assure participants' anonymity; Y = yes; N = no; NR = Not reported; BMI (body mass index) = categorised with reference to WHO international classification guidelines (normal weight: 18.9–24.9 kg/m2, overweight: 25.0–29.9 kg/m2, obese: ≥30.0 kg/m2)[20]; Joint pain = 'Do you experience pain, discomfort, or have a problem with your: hip(s) or groin, knee(s), ankle(s), spine (back or neck), shoulder(s), elbow(s), wrist(s), finger(s) or hand(s)'; OA (osteoarthritis) = 'Have you ever been told you have wear and tear, degeneration or osteoarthritis by a doctor?'; Past injury = 'Have you ever had any injuries leading to more than 4 weeks of reduced participation in exercise, training or sport?'; Arthroscopic joint surgery = one or more arthroscopic joint surgery; Joint replacement = have you ever had joint replacement surgery?; UK professional seasons = number of seasons playing professional cricket in the UK; Considering the benefits and risks of cricket I would do the same again = Considering the benefits and risks of my previous



For peer review only - http://bmjopen.bmj.com/site/about/guidelines.xhtml

participation in cricket, I would do the same again (responses on a 5 point Likert-scale: strongly agree, agree, undecided, disagree, strongly disagree); I would recommend cricket to significant others = Considering the benefits and risks of my previous participation in cricket, I would recommend this to my children, relatives or close friends (responses on a 5 point Likert-scale: strongly agree, agree, undecided, disagree, strongly disagree)

For peer review only



For peer review only - http://bmjopen.bmj.com/site/about/guidelines.xhtml

## Figure legends

**Figure 1** A conceptual flow diagram summarising main findings and potential interactions between key themes

Green arrows and boxes represent factors with potential to *positively* impact quality of life;
Red arrows and boxes represent factors with potential to *negatively* impact quality of life;
A. Reflecting upon positive experiences in cricket and psychological attributes may positively impact quality of life following retirement from professional cricket;
B. Reflecting upon positive experiences in cricket and psychological attributes may reduce the impact of pain, osteoarthritis and physical limitations on quality of life after retirement from professional cricket;
Together, A. and B. provide one potential explanation for high quality of life despite a high prevalence of pain, osteoarthritis and physical limitations in this sample of retired professional cricketers;
Question marks denote uncertainty surrounding the nature of the relationship between cricket-related experiences and psychological attributes common in successful cricketers, since individuals may possess these attributes prior to cricket participation

For peer review only



For peer review only - http://bmjopen.bmj.com/site/about/guidelines.xhtml

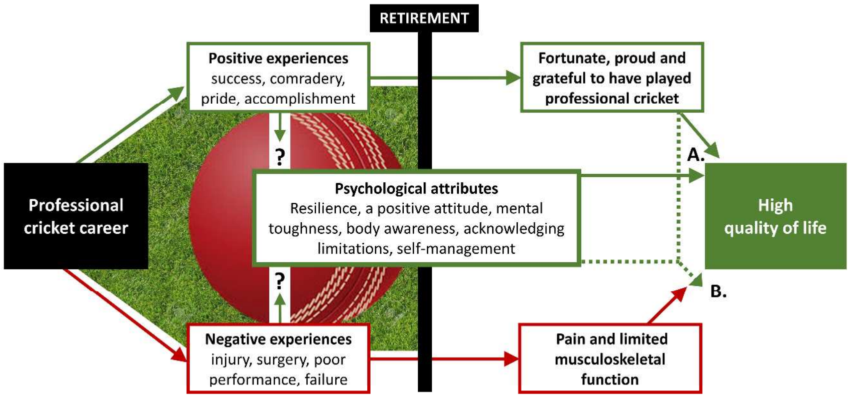


Figure 1 A conceptual flow diagram summarising main findings and potential interactions between key themes├ ┬ Green arrows and boxes represent factors with potential to positively impact quality of life;
Red arrows and boxes represent factors with potential to negatively impact quality of life;
A. Reflecting upon positive experiences in cricket and psychological attributes may positively impact quality of life following retirement from professional cricket;
B. Reflecting upon positive experiences in cricket and psychological attributes may reduce the impact of pain, osteoarthritis and physical limitations on quality of life after retirement from professional cricket;
Together, A. and B. provide one potential explanation for high quality of life despite a high prevalence of pain, osteoarthritis and physical limitations in this sample of retired professional cricketers;
Question marks denote uncertainty surrounding the nature of the relationship between cricket-related experiences and psychological attributes common in successful cricketers, since individuals may possess these attributes prior to cricket participation

677x381mm (300 x 300 DPI)

only

**Supplementary Appendix 1** Semi-structured interview guide

| |
|---|
| 1. Can you describe any physical activity, exercise or sport that you currently take part in? |
| 2. Has that remained fairly constant since you retired from cricket or has it changed over the years? |
| 3. Have you played cricket again since retiring? Why/why not? |
| 4. What was your motivation for playing cricket? |
| 5. Are you as physically active as you would like to be? If no, why not? How does this make you feel? |
| 6. What is your motivation for taking part in physical activity/exercise/sport? |
| 7. How important is being physically active to you? (If important, why is it important? / If not important, has it always been this way?) |
| 8. Does the type of physical activity that you do matter to you, or would you be satisfied taking part in any form of physical activity?<br>(ask about specific forms of exercise that they find *dissatisfying* and why) |
| 9. What physical activity goals are you currently trying to achieve, if any? |
| 10. What are the barriers or challenges, if any, that impact on your ability to be physically active? |
| 11. Do you think that retired cricketers face the same challenges with being physically active as the general population, or are they unique or different in some way? |
| 12. Some retired cricketers become physically inactive, what advice would you give to help them maintain a physically active lifestyle after retiring from cricket? |
| 13. If you wanted to increase your physical activity levels, what do you think would help you to do so? |
| 14. Can you describe any positive or negative impacts that your previous participation in cricket has had upon your current physical activity patterns? |
| 15. If you hadn't played professional cricket, do you think that you would be more or less active, than you currently are? |
| 16. Does your current ability to participate in physical activity impact upon your quality of life? If yes, in what ways? If no, why not? |
| 17. Overall how satisfied are you with your current quality of life?<br>Do you think that this is related to your past career in cricket? |
| 18. Is there anything more you would like to add about your experiences with physical activity after retiring from professional cricket? |

## COREQ (COnsolidated criteria for REporting Qualitative research) Checklist

A checklist of items that should be included in reports of qualitative research. You must report the page number in your manuscript where you consider each of the items listed in this checklist. If you have not included this information, either revise your manuscript accordingly before submitting or note N/A.

| Topic | Item No. | Guide Questions/Description | Reported on Page No. |
|---|---|---|---|
| **Domain 1: Research team and reflexivity** | | | |
| *Personal characteristics* | | | |
| Interviewer/facilitator | 1 | Which author/s conducted the interview or focus group? | 8 |
| Credentials | 2 | What were the researcher's credentials? E.g. PhD, MD | 8 |
| Occupation | 3 | What was their occupation at the time of the study? | 18 |
| Gender | 4 | Was the researcher male or female? | 8 |
| Experience and training | 5 | What experience or training did the researcher have? | 8, 18 |
| *Relationship with participants* | | | |
| Relationship established | 6 | Was a relationship established prior to study commencement? | 8 |
| Participant knowledge of the interviewer | 7 | What did the participants know about the researcher? e.g. personal goals, reasons for doing the research | 7, 8 |
| Interviewer characteristics | 8 | What characteristics were reported about the inter viewer/facilitator? e.g. Bias, assumptions, reasons and interests in the research topic | 7, 18 |
| **Domain 2: Study design** | | | |
| *Theoretical framework* | | | |
| Methodological orientation and Theory | 9 | What methodological orientation was stated to underpin the study? e.g. grounded theory, discourse analysis, ethnography, phenomenology, content analysis | 9 |
| *Participant selection* | | | |
| Sampling | 10 | How were participants selected? e.g. purposive, convenience, consecutive, snowball | 7 |
| Method of approach | 11 | How were participants approached? e.g. face-to-face, telephone, mail, email | 7 |
| Sample size | 12 | How many participants were in the study? | 8 |
| Non-participation | 13 | How many people refused to participate or dropped out? Reasons? | 8 |
| *Setting* | | | |
| Setting of data collection | 14 | Where was the data collected? e.g. home, clinic, workplace | 8 |
| Presence of non-participants | 15 | Was anyone else present besides the participants and researchers? | 8 |
| Description of sample | 16 | What are the important characteristics of the sample? e.g. demographic data, date | 10, 11 |
| *Data collection* | | | |
| Interview guide | 17 | Were questions, prompts, guides provided by the authors? Was it pilot tested? | 9 |
| Repeat interviews | 18 | Were repeat inter views carried out? If yes, how many? | 18 |
| Audio/visual recording | 19 | Did the research use audio or visual recording to collect the data? | 8 |
| Field notes | 20 | Were field notes made during and/or after the inter view or focus group? | 9 |
| Duration | 21 | What was the duration of the inter views or focus group? | 8 |
| Data saturation | 22 | Was data saturation discussed? | 8 |
| Transcripts returned | 23 | Were transcripts returned to participants for comment and/or | 18 |

For peer review only

| Topic | Item No. | Guide Questions/Description | Reported on Page No. |
|---|---|---|---|
| | | correction? | |
| **Domain 3: analysis and findings** | | | |
| *Data analysis* | | | |
| Number of data coders | 24 | How many data coders coded the data? | 10 |
| Description of the coding tree | 25 | Did authors provide a description of the coding tree? | 10 |
| Derivation of themes | 26 | Were themes identified in advance or derived from the data? | 9 |
| Software | 27 | What software, if applicable, was used to manage the data? | 9 |
| Participant checking | 28 | Did participants provide feedback on the findings? | 18 |
| *Reporting* | | | |
| Quotations presented | 29 | Were participant quotations presented to illustrate the themes/findings? Was each quotation identified? e.g. participant number | 11-15 |
| Data and findings consistent | 30 | Was there consistency between the data presented and the findings? | 11-15 |
| Clarity of major themes | 31 | Were major themes clearly presented in the findings? | 11-15 |
| Clarity of minor themes | 32 | Is there a description of diverse cases or discussion of minor themes? | 12 |

Developed from: Tong A, Sainsbury P, Craig J. Consolidated criteria for reporting qualitative research (COREQ): a 32-item checklist for interviews and focus groups. *International Journal for Quality in Health Care*. 2007. Volume 19, Number 6: pp. 349 – 357

**Once you have completed this checklist, please save a copy and upload it as part of your submission. DO NOT include this checklist as part of the main manuscript document. It must be uploaded as a separate file.**

For peer review only
